# Mental health and access to support among Latin Americans in the United Kingdom: A scoping review

**DOI:** 10.1016/j.jmh.2026.100457

**Published:** 2026-10-29

**Authors:** Rhysha Gallo, María Camila García Durán, Vaughan Bell, Norha Vera San Juan

**Affiliations:** a Clinical, Educational and Health Psychology, University College London, Gower Street, London WC1E 6BT, UK; b Centre for Global Mental Health, Health Service and Population Research Department, Institute of Psychiatry, Psychology and Neuroscience, King’s College London, De Crespigny Park, London SE5 8AF, UK; c Salud Poblacional, Fundación Santa Fe de Bogotá, Carrera 7 No. 117-15, Bogotá, Colombia; d South London and Maudsley NHS Foundation Trust, Maudsley Hospital, Denmark Hill, London, SE5 8AZ, UK; e Department of Health Services and Population Research, King’s College London, De Crespigny Park, London SE5 8AF, UK; f Department of Public Health and Primary Care, University College London, Gower Street, London WC1E 6BT, UK

**Keywords:** Latin Americans, Migration, Mental health, Healthcare access, Scoping review, Help-seeking

## Abstract

**Background:**

Latin Americans in the UK are a diverse population whose mental health needs are shaped by migration histories, settlement and structural constraints, yet remain under-recognised within research, policy and routine data collection. To date, there has been no systematic synthesis of evidence relating to mental health and experiences of seeking mental health support among this community.

**Methods:**

A scoping review examined mental health needs, psychological wellbeing and experiences of accessing formal and informal mental health support among Latin Americans in the UK.

**Results:**

Twenty-four publications met inclusion criteria following searches across PsycINFO, MEDLINE and EMBASE and grey literature searching. Psychological distress was associated with migration-related stress, insecure employment, housing precarity, discrimination, immigration insecurity and obstacles to accessing care. Wellbeing was linked to belonging, social connection, cultural continuity and trusted community spaces. Barriers to support included language difficulties, limited service awareness, long waiting times and concerns regarding culturally responsive care. Community organisations and informal networks were frequently described as sources of advocacy, navigation and emotional support.

**Conclusions:**

The reviewed literature suggests that mental health and help-seeking among Latin Americans in the UK are closely linked to labour and housing precarity, immigration insecurity, language barriers and difficulties accessing care. Community organisations and informal networks emerged as important sources of support. Findings highlight the potential value of culturally responsive and language-concordant care, clearer access pathways and stronger partnerships with trusted community organisations. Greater visibility within health research, policy and routine data systems may help identify unmet needs and inform efforts to reduce inequalities in access to mental health support.

## Highlights

• Latin Americans remain under-recognised within UK mental health research.
• Migration-related stressors shape mental health and wellbeing.
• Language and cultural barriers affect access to mental health support.
• Community organisations play key roles in help-seeking pathways.
• More culturally responsive mental health provision is needed.

## 1 Background

Latin Americans represent a sizeable and increasingly visible population in the United Kingdom, including migrants and people of Latin American heritage across generations, yet remain relatively under-recognised within national statistics, health research and policy frameworks. The term “Latin American” is complex and contested. Definitions of “Latin America” vary across disciplinary and geopolitical contexts, reflecting ambiguity in how both “Latin” and “America” are delimited (Durand and Massey, 2010). In some contexts, Latin America is understood primarily as a geographical region, while in others it functions as a broader cultural, linguistic, diasporic or political category.

This definitional ambiguity is compounded by the heterogeneity of the population itself. “Latin American” should not be understood as a homogeneous identity. The category encompasses people from more than 20 countries with distinct national histories, racialised identities, languages, migration pathways, legal statuses, socio-economic positions and relationships to UK institutions. Health research on Latin American populations has similarly cautioned that broad umbrella categories can obscure important within-group differences, including differences by national origin, nativity, race, language and socio-economic position (Aragones et al., 2014; Errisuriz et al., 2024).

Although 2021 Census data recorded 277,803 residents born in Central or South America in England and Wales, including over 150,000 in London (Office for National Statistics, 2023), these figures are likely to underestimate the Latin American population because “Latin American” is not routinely captured as a distinct ethnic category and the estimates exclude UK-born Latin Americans. Consequently, many Latin Americans are subsumed within broad residual categories such as “Any Other Ethnic Group”, limiting visibility within health and administrative datasets and making it difficult to identify population needs or inequalities in service access (Healthwatch Southwark, 2023; McIlwaine and Bunge, 2016).

This statistical invisibility sits alongside substantial diversity in migration route, legal status and generation. Earlier research in London identified a population including regular, irregular and second-generation Latin Americans, with substantial concentration in the capital and particularly large Brazilian and Colombian-origin communities (McIlwaine et al., 2011). Subsequent work has highlighted onward migration from Southern Europe, including Latin Americans who moved to the UK after periods of residence in Spain, Portugal or Italy and whose rights and sense of belonging were reshaped by Brexit and the EU Settlement Scheme (Mas Giralt and Granada, 2015; McIlwaine and Bunge, 2016; Turcatti and Vargas-Silva, 2021; Turcatti and Vargas-Silva, 2022). The population also includes people with insecure or undocumented status, people seeking asylum, refugees and UK-born descendants of migrants, although these groups are not always clearly distinguished within the available evidence. These migration profiles may shape exposure to different structural conditions, including entitlement uncertainty, employment precarity, housing insecurity, immigration enforcement, welfare eligibility and access to community support.

These demographic and structural conditions are important because migration is widely recognised as a social determinant of mental health. Although migration may create opportunities for safety, education, employment and social mobility, it can also involve social disruption, family separation, discrimination, financial insecurity and adaptation to unfamiliar social systems (Bhugra, 2004). For Latin Americans in the UK, these wider migration-related determinants intersect with labour market disadvantage, housing insecurity, language barriers, discrimination and uncertainty regarding immigration status (McIlwaine et al., 2011; Montañez, 2020; Southwark Council, 2024). These experiences have been associated with depression, anxiety, trauma-related distress and social isolation, particularly among migrants facing labour exploitation, economic insecurity or discrimination (de la Silva et al., 2019). Alongside these challenges, wellbeing may also be shaped by belonging, cultural identity and access to supportive family, faith, peer and community networks (McIlwaine et al., 2011; Turcatti and Vargas-Silva, 2021).

Access to formal mental health support is shaped by both general and UK-specific barriers. Existing evidence suggests that many Latin Americans encounter language difficulties, uncertainty regarding eligibility for care, limited awareness of available services and experiences of culturally insensitive or discriminatory care (Healthwatch Southwark, 2023; Rassa et al., 2023). Some individuals may therefore delay help-seeking or rely more heavily on informal, community-based or transnational sources of support (Healthwatch Southwark, 2023; Montañez, 2020; Turcatti and Vargas-Silva, 2021).

These access barriers are shaped by the legal, healthcare and welfare context in which Latin Americans in the UK seek support. In England, formal mental health support is often organised through the National Health Service (NHS), alongside local authority, voluntary-sector and community-based provision, which may be difficult to navigate for people unfamiliar with the system or facing language barriers (Healthwatch Southwark, 2023; Southwark Council, 2024). Although GP registration should not require proof of address, identity or immigration status, uncertainty about entitlement, NHS charging rules and possible data-sharing may deter some migrants from seeking care (GOV.UK, 2023; NHS, 2025; Rassa et al., 2023). Wider immigration and welfare policies, including no recourse to public funds, may also shape mental health indirectly by restricting access to housing, welfare support and financial safety nets (House of Commons Library, 2025; Lacey, 2024; Migration Observatory, 2023). For Latin Americans in the UK, these dynamics are further shaped by onward migration from Europe and the post-Brexit restructuring of rights and belonging (Mas Giralt and Granada, 2015; Turcatti and Vargas-Silva, 2021; Turcatti and Vargas-Silva, 2022). A UK-specific synthesis is therefore needed to understand how migration-related stressors intersect with NHS access routes, immigration policy, welfare eligibility and community-sector support.

Existing evidence relating to the mental health of Latin Americans in the UK remains fragmented despite increasing recognition of migrant mental health as a public health priority. Much of the literature consists of organisational reports, local needs assessments and other forms of grey literature rather than peer-reviewed academic research. In addition, Latin Americans are often grouped within broader ethnic categories in UK research, which may obscure the specific social, cultural and structural contexts shaping mental health and help-seeking within this population. To date, there has been no systematic synthesis specifically examining mental health, psychological wellbeing and experiences of accessing mental health support among Latin Americans in the UK.

This scoping review addressed two questions: (1) what is known about the mental health needs and psychological wellbeing of Latin Americans in the UK and (2) what is known about how Latin Americans access and experience formal and informal sources of mental health support. By mapping the available evidence, the review sought to identify key themes, highlight gaps in knowledge and inform future research, policy and practice relating to the mental health of Latin Americans in the UK.

## 2 Methods

### 2.1 Study design

A scoping review was conducted to map evidence relating to the mental health needs, psychological wellbeing and experiences of accessing mental health support among Latin Americans living in the UK. This design was appropriate given the exploratory topic and heterogeneous evidence base, which spans qualitative research, community reports, local needs assessments and studies in which Latin Americans are included within broader migrant or racially minoritised populations. The review followed Arksey and O’Malley’s (2005) framework, refined by Levac et al. (2010) and reporting adhered to PRISMA-ScR guidance (Tricco et al., 2018).

Consistent with migration-health research, the review adopted a broad conceptualisation of mental health and wellbeing encompassing psychological distress, emotional wellbeing, belonging and unmet support needs (Keyes, 2002). Formal support included statutory and clinical services, such as NHS mental health services, primary care and private providers, while informal support included community organisations, peer networks, faith groups and other non-statutory sources of support.

### 2.2 Search strategy

Searches were structured around three concepts: Latin Americans, the United Kingdom and mental health/support access. Keywords within each category were combined using Boolean operators. Searches were conducted between September and November 2025 across PsycINFO, MEDLINE and EMBASE. The full search strategy is presented in Appendix A.

Grey literature searching was included because preliminary scoping indicated that much of the evidence relating to Latin Americans in the UK has been produced by community organisations, local authorities, advocacy groups and service providers rather than academic publishers. Although structured grey literature databases may have identified additional indexed sources, Google Advanced Search, targeted organisational website searching and citation tracking were used to identify community-based and practice-based evidence that may not be indexed in bibliographic databases. Google Advanced Search used combinations of population, setting and mental health/support access terms to identify publicly available organisational reports, local needs assessments and community research outputs. The full grey literature search strategy is presented in Appendix A. The first 100 results for each search string were screened and potentially relevant records were saved with the search date, title, organisation and URL.

### 2.3 Inclusion criteria

Publications were eligible for inclusion if they focused on Latin Americans living in the UK and examined mental health needs, psychological wellbeing and/or experiences of accessing or seeking formal or informal mental health support. For eligibility purposes, “Latin American” was operationalised pragmatically, reflecting its use in UK community, policy and research contexts rather than implying a single homogeneous identity. It referred to individuals with origins in Latin America, including South America, Central America and Mexico, residing in the UK and included both Spanish- and Portuguese-speaking populations. Mexico was included because the term was used as a diasporic, cultural and migration-related category rather than a strictly continental classification; although geographically located in North America, Mexico is commonly included within Latin American classifications because of shared historical, linguistic, cultural and geopolitical connections with the wider region (Durand and Massey, 2010). Studies involving broader migrant or racially minoritised samples were eligible only where findings related specifically to Latin Americans could be distinguished from those of other groups.

Given the relationship between social adversity and psychological wellbeing, studies examining welfare, legal, housing or community support were included where findings related to mental health or distress. Qualitative, quantitative and mixed-methods studies were eligible. Both academic and grey literature were included; however, only publications reporting primary empirical data were eligible. Primary empirical data were defined as findings generated through direct data collection, including interviews, focus groups, surveys, consultations, community workshops, casework data or local needs assessments. For grey literature, reports were eligible where they presented methods and findings based on data collected from Latin American participants, community members, service users, practitioners or stakeholders. Publications were excluded where they consisted only of advocacy statements, policy commentary, service descriptions, literature reviews, webpages, opinion pieces or reports that discussed Latin Americans without presenting new data or distinguishable findings relevant to the review questions.

Publications were excluded if they neither included Latin American participants nor presented original empirical findings specifically concerning Latin Americans, if findings relating to Latin Americans could not be distinguished from broader ethnic categories or if the research was conducted outside the UK. No language or publication date restrictions were applied.

### 2.4 Source selection

Records were imported into Rayyan, a web-based systematic review management platform (Ouzzani et al., 2016) and duplicates were removed. Title and abstract screening was conducted by the lead reviewer (RG) using predefined eligibility criteria. A second reviewer (MCG) independently screened a random sample of 10% of records. Discrepancies were resolved through discussion with a third reviewer (NVSJ). The screening and study selection process is presented in the PRISMA-ScR flow diagram (Fig. 1).

**Fig. 1:**
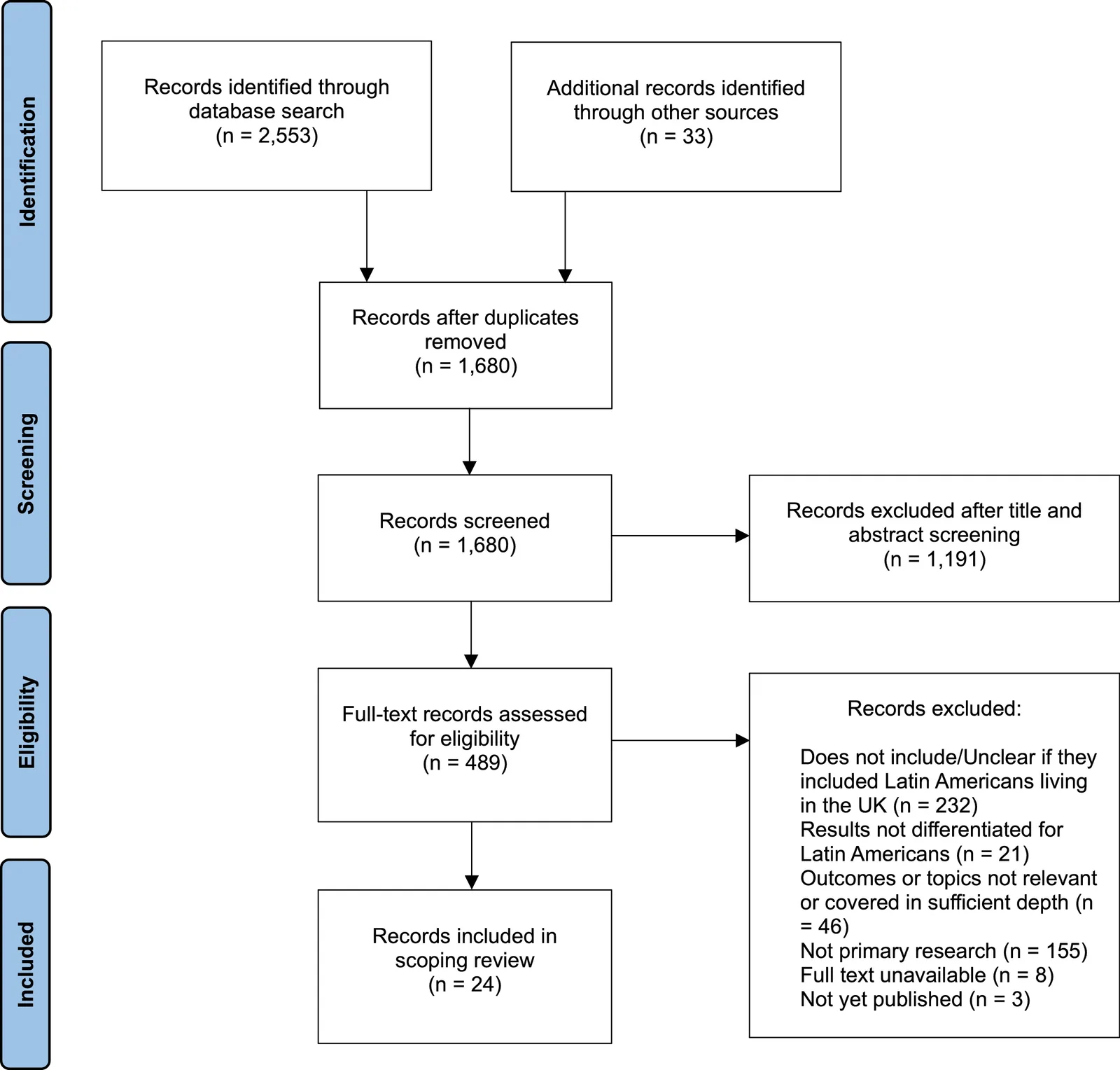
PRISMA-ScR Flow Diagram.

### 2.5 Data extraction and synthesis

Data were extracted using a structured Excel spreadsheet based on a predefined charting framework, which is presented in Appendix D. Extracted information included publication, methodological and participant characteristics; findings relating to mental health, psychological wellbeing and support access; whether publications focused specifically on Latin Americans or included them within broader multi-ethnic samples; and whether Latin American-specific findings were reported separately. Data extraction was conducted by the lead reviewer.

Formal critical appraisal of peer-reviewed studies was not undertaken, consistent with scoping review methodology (Arksey and O’Malley, 2005; Levac et al., 2010). However, grey literature was appraised using selected AACODS domains (Tyndall, 2010), focusing on authority, accuracy and objectivity. The grey literature appraisal framework and source-level appraisal are presented in Appendix E.

Findings were synthesised descriptively and thematically. Source characteristics were first summarised descriptively and findings relevant to the review questions were charted in the extraction spreadsheet. The lead reviewer then coded extracted findings inductively and grouped similar findings into preliminary subthemes. Subthemes were compared across publications and refined into broader themes within two analytic domains derived from the review questions: (1) mental health needs and psychological wellbeing and (2) experiences of accessing and seeking mental health support. The thematic charting framework is presented in Appendix D. Preliminary themes were reviewed by NVSJ and discussed in supervision meetings. Comments, uncertainties and disagreements regarding theme boundaries, interpretation or organisation were resolved through discussion and used to refine the final themes.

### 2.6 Researcher position and reflexivity

Given the interpretive nature of thematic synthesis, reflexivity was considered throughout the analytic process. Data extraction, charting and initial coding were conducted by RG, a trainee clinical psychologist with a professional interest in accessibility and equity within mental health services. RG does not identify as Latin American and therefore occupied a primarily outsider position in relation to the population of interest, while also holding partial insider positions through personal and professional experience of mental health services.

These positions may have shaped the synthesis in different ways. Professional experience may have increased sensitivity to structural barriers, service accessibility and help-seeking processes, while also introducing assumptions about how services operate and how people navigate support. To support reflexivity, emerging interpretations, theme boundaries and analytic decisions were discussed in supervision with NVSJ and with the wider review team, which included researchers with experience in migration, mental health and Latin American community contexts. These discussions supported critical reflection on the risk of over-interpreting findings, privileging service or community-sector perspectives, or treating Latin Americans as a homogeneous group. Themes were therefore developed as interpretive but source-grounded accounts of the included literature.

## 3 Results

Academic database searches yielded 2,553 records, with an additional 33 records identified through grey literature searches and citation tracking. After removing 906 duplicates, 1,680 records remained for title and abstract screening. Twenty-four publications met the final inclusion criteria.

### 3.1 Literature characteristics

A total of 24 publications were included, comprising 10 academic publications and 14 grey literature reports produced by community organisations, local authorities and research organisations. Publications were published between 2010 and 2025 and were predominantly qualitative or mixed-methods in design. The evidence base was predominantly London-focused, although several publications drew on wider UK populations and broader geographical contexts.

The evidence base primarily examined the experiences of Latin American migrants living in the UK, including specific subgroups such as migrant women, trans and non-binary migrants, men who have sex with men, older adults and family carers. However, studies varied in the extent to which they disaggregated findings by migration route, generation, nationality, language or legal status. Across the literature, mental health and wellbeing were rarely examined in isolation from migration experiences. Instead, distress and wellbeing were typically discussed within broader contexts of migration, employment, housing, discrimination, service access and community life. Characteristics of included publications are presented in Table 1.

**Table 1:** Characteristics of included literature (n=24).

*Title*	*Author*	*Year*	*Location*	*Type*	*Method*	*Population*	*Key Focus*
Trans and Non-Binary Latin American Migrants in London: An Intersectional Analysis of Migration and Psychological Well-Being	Cordoba et al.	2025	London	Academic article	Qualitative	Trans and non-binary Latin American migrants	Migration, gender identity and wellbeing
HIV and migration: Understanding the barriers faced by people born abroad living with HIV in the UK	National AIDS Trust	2021	London	Community research report	Participatory qualitative	Migrants living with HIV, including Latin American participants	HIV care access, migration-related barriers and wellbeing
Migrant digital placemaking for wellbeing: A netnography of Facebook groups	te Lintelo et al.	2024	London	Academic article	Qualitative (netnography)	Latin American migrants using Facebook groups	Digital communities, belonging and wellbeing
Are low-intensity CBT interventions effective and meaningful for the Latino community in the UK?	Lopez et al.	2014	London	Academic article	Mixed methods	First-generation Latin American migrants	Effectiveness and acceptability of low-intensity CBT
The Role of Attachment in Immigrant Sociocultural Adaptation and Psychological Distress	Sochos and Diniz	2012	UK (London heavy focus)	Academic article	Quantitative	Brazilian migrants living in the UK	Attachment, adaptation and distress
Determinants of substance use amongst Brazilians residing in the UK: The role of acculturation	Canfield et al.	2017	UK–Brazil	Academic article	Mixed methods (comparative study)	Brazilian adults in the UK and Brazil	Migration, acculturation and substance use
Barriers and facilitators to addressing mental health needs among Asian, Black and Latin American men who have sex with men (MSM)	Ghobrial et al.	2025	England–Wales	Academic article	Qualitative (comparative study)	Asian, Black and Latin American cis-gender MSM from racially minoritised groups	Mental health needs, stigma, coping, barriers and facilitators to accessing support
Experience of UK Latin Americans caring for a relative living with dementia	Guerra et al.	2022	UK-wide	Academic article	Qualitative	Latin American family carers or relatives living with dementia	Family caregiving experiences, cultural understandings of dementia and access to support
Migrant machismos: exploring gender ideologies and practices among Latin American migrants in London from a multi-scalar perspective	McIlwaine	2010	London	Academic article	Qualitative	Latin American migrants in London	Gender, migration and wellbeing
Constructing Migrant Wellbeing: An Exploration of Life Satisfaction Amongst Peruvian Migrants in London	Wright	2011	London	Academic article	Mixed methods (qualitative dominant)	Peruvian migrants in London	Migration, wellbeing and social inclusion
Gender Transformations and Gender-Based Violence among Latin American Migrants in London	McIlwaine and Carlisle	2011	London	Book chapter	Qualitative	Latin American migrants in London (focus on women affected by gender-based violence)	Gender-based violence and migration
Access to Health and Social Care Services for Latin American Communities in Southwark	Healthwatch Southwark	2023	London	Needs assessment report	Mixed methods	Latin American residents	Healthcare barriers and service access
Latin American Health - Southwark’s Joint Strategic Needs Assessment	Southwark Council	2024	London	Needs assessment report	Mixed methods	Latin American residents and community stakeholders in Southwark	Health inequalities and service access
Written evidence on COVID-19 impacts on Latin Americans in the UK	CLAUK and LAWRS	2020	UK (London heavy focus)	Parliamentary evidence	Mixed methods and evidence synthesis	Latin American migrants in the UK	COVID-19, wellbeing and service access
Listening Project: Equity in mental health support for racially minoritised young people	Transformation Partners in Health and Care	2024	London	Community research report	Qualitative	Racially minoritised young people in London, including Latin American participants	Mental health inequalities and culturally responsive care
Lemme Hablar: Community Engagement and the Latin American Community in Southwark	Montañez	2020	London	Community research report	Qualitative	Latin American residents in Southwark and Lambeth	Community needs and local service engagement
Latin Americans in Southwark: Service access and provision	Berg (COMPAS, University of Oxford)	2017	Lon don	Needs assessment report	Mixed methods	Service providers working with Latin American communities in Southwark	Structural barriers and support access
Access to Primary Care and Social Care Services in Islington for Latin American over 50s	Healthwatch Islington (LAWRS, CLAUK)	2016	London	Needs assessment report	Mixed methods	Latin Americans aged 50+ in Islington	Primary care and social care access
The Experiences of London’s Latin American Migrants during Brexit and the COVID-19 Pandemic	Turcatti and Vargas-Silva (COMPAS, LAH, University of Oxford)	2021	London	Community research report	Qualitative	Latin American migrants and students in London and NGO/community workers	Brexit, COVID-19 and wellbeing
The Unheard Workforce: Experiences of Latin American migrant women in cleaning, hospitality and domestic work	de la Silva et al. (LAWRS, Trust for London)	2019	London	Community research report	Mixed methods	Latin American migrant women in hospitality and domestic work	Labour exploitation, wellbeing and support access
Towards Visibility: The Latin American community in London	McIlwaine and Bunge (QMUL, LAWRS, Trust for London)	2016	London	Needs assessment report	Mixed methods	Latin American migrants in London (focus on onward migrants from Europe)	Housing, employment and healthcare access
No Longer Invisible: The Latin American community in London	McIlwaine et al. (QMUL, LAWRS, Trust for London)	2011	London	Needs assessment report	Mixed methods	Latin American migrants (focus on second-generation Latin Americans in London)	Wellbeing, discrimination and service access
The Right to Healthcare: A community-led approach to better health outcomes for the Latin American community	Caicedo (LAWRS, IRMO)	2023	London	Needs assessment report	Mixed methods	Latin American residents in Southwark	Migration status, inequality and healthcare access
Supporting Brazilian Women in London: Service Provision for Survivors of Violence Against Women and Girls	Evans and McIlwaine (QMUL)	2017	London	Community research report	Qualitative	Brazilian women in London affected by VAWG and service providers	Violence, migration and support needs

### 3.2 Mental health needs and psychological wellbeing

#### 3.2.1 Structural factors associated with psychological distress

Across the literature, psychological distress among Latin Americans in the UK was closely linked to structural conditions associated with migration and settlement, including precarious employment, housing insecurity, immigration-related uncertainty and discrimination (Caicedo, 2023; Canfield et al., 2017; McIlwaine, 2010; Sochos and Diniz, 2012; Wright, 2011).

Participants were frequently described as working in low-paid and insecure sectors such as cleaning, hospitality and domestic labour despite previous qualifications or professional experience (de la Silva et al., 2019; McIlwaine and Bunge, 2016; McIlwaine et al., 2011). Downward occupational mobility, exploitative conditions and financial insecurity were associated with chronic stress, anxiety, reduced self-worth and, among women affected by workplace abuse or violence, fear, hopelessness and trauma-related distress (Caicedo, 2023; Coalition of Latin American Organisations in the UK, Latin American Women’s Rights Service, 2020; de la Silva et al., 2019; McIlwaine, 2010).

Housing insecurity, overcrowding and unstable accommodation were similarly described as contributing to stress, particularly when combined with low income or insecure immigration status (Cordoba et al., 2025; McIlwaine and Carlisle, 2011, McIlwaine et al., 2011). Immigration-related stressors, including fear of deportation, legal uncertainty and restrictions associated with no recourse to public funds (NRPF), were associated with anxiety, reduced safety and mistrust of public services (Coalition of Latin American Organisations in the UK, Latin American Women’s Rights Service, 2020; Cordoba et al., 2025; Healthwatch Southwark, 2023; McIlwaine and Carlisle, 2011, McIlwaine et al., 2011; National AIDS Trust, 2021).

Experiences of racism, xenophobia and exclusion were also linked to emotional distress, reduced self-esteem and feelings of not belonging (Caicedo, 2023; Healthwatch Southwark, 2023; Lopez et al., 2014; McIlwaine et al., 2011). Among trans and non-binary Latin American migrants, intersecting experiences of ethnic and gender-based discrimination compounded psychological distress (Cordoba et al., 2025).

#### 3.2.2 Migration, identity and belonging

Migration experiences were closely linked to psychological wellbeing. Migration, settlement and adaptation were frequently described as involving loss, uncertainty, family separation and social disruption (Caicedo, 2023; McIlwaine and Bunge, 2016; McIlwaine et al., 2011; Turcatti and Vargas-Silva, 2021). Feelings of loneliness and social disconnection were common, particularly among migrants with limited social networks or difficulties integrating into wider society (Caicedo, 2023; McIlwaine and Carlisle, 2011; te Lintelo et al., 2024).

Belonging and exclusion emerged as important dimensions of wellbeing. Participants described navigating identities shaped by nationality, language, ethnicity and migration status within the UK context, while legal uncertainty, discrimination and perceived exclusion from British society undermined feelings of safety and belonging (Caicedo, 2023; Canfield et al., 2017; Cordoba et al., 2025; McIlwaine, 2010; McIlwaine et al., 2011). Among sexual and gender minority participants, belonging was also shaped by acceptance within both Latin American and LGBTQ+ communities (Cordoba et al., 2025).

Acculturative stress linked to unfamiliar systems, language environments and social expectations was also associated with distress (Canfield et al., 2017; Evans and McIlwaine, 2017; Ghobrial et al., 2025). Conversely, cultural continuity through shared language, community networks, online groups and culturally meaningful spaces supported connection, identity affirmation and belonging (Guerra et al., 2022; Montañez, 2020; te Lintelo et al., 2024).

#### 3.2.3 Gendered and intersectional experiences of distress

Mental health experiences were shaped by intersecting inequalities, including gender, sexuality, immigration status and labour market position (Cordoba et al., 2025, Ghobrial et al., 2025; McIlwaine, 2010; McIlwaine and Carlisle, 2011). Research focusing on Latin American migrant women described the psychological impacts of labour exploitation, economic insecurity and gender-based violence, particularly where abusive working conditions, immigration insecurity, financial dependency and limited access to welfare or housing support intersected (Coalition of Latin American Organisations in the UK, Latin American Women’s Rights Service, 2020; Cordoba et al., 2025; de la Silva et al., 2019; McIlwaine et al., 2011; Evans and McIlwaine, 2017). Immigration status was also described as being used as a tool of control within abusive relationships, restricting some women’s ability to seek help or leave harmful situations (Coalition of Latin American Organisations in the UK, Latin American Women’s Rights Service, 2020; Evans and McIlwaine, 2017, McIlwaine and Carlisle, 2011).

Other studies highlighted the experiences of Latin American trans and non-binary migrants and men who have sex with men. Stigma, discrimination and minority stress linked to gender identity or sexuality compounded migration-related stressors and contributed to psychological distress, including through transphobia, xenophobia, family rejection and social isolation (Cordoba et al., 2025; Ghobrial et al., 2025). Cultural expectations around masculinity, self-reliance and emotional restraint also shaped wellbeing and help-seeking among some Latin American men, particularly following migration-related role changes or status loss (Ghobrial et al., 2025; McIlwaine, 2010).

#### 3.2.4 Relational and community dimensions of wellbeing

Alongside experiences of distress, the literature highlighted the importance of social relationships, community networks and collective spaces in supporting wellbeing (Caicedo, 2023, Guerra et al., 2022, McIlwaine et al., 2011, Montañez, 2020). Family relationships, faith communities, peer networks, online groups and transnational family ties were described as providing emotional support, practical assistance and a sense of belonging, although separation from family could also contribute to vulnerability and loneliness (Caicedo, 2023, Healthwatch Southwark, 2023, McIlwaine et al., 2011, Turcatti and Vargas-Silva, 2021).

Community organisations were consistently described as important sources of support and wellbeing. Organisations including LAWRS, IRMO, CLAUK and local Latin American community centres fostered wellbeing through culturally familiar and linguistically accessible spaces for connection, peer support, advocacy and practical assistance (Healthwatch Southwark, 2023; McIlwaine et al., 2011; Montañez, 2020). Shared language, particularly Spanish and Portuguese, facilitated trust, emotional expression and meaningful communication, while culturally affirming spaces supported identity, safety and belonging (Cordoba et al., 2025; Guerra et al., 2022; Lopez et al., 2014, Montañez, 2020, te Lintelo et al., 2024).

### 3.3 Experiences of accessing mental health support

#### 3.3.1 Structural barriers to formal support

Structural barriers within health and social care systems were frequently identified as obstacles to accessing mental health support (Berg, 2017; McIlwaine and Carlisle, 2011; Southwark Council, 2024; Transformation Partners in Health and Care, 2024). Participants described long waiting times, complex or fragmented care pathways, uncertainty regarding referral routes and limited awareness of available services (Healthwatch Islington, 2016; Healthwatch Southwark, 2023; Montañez, 2020). Some individuals described delays, redirection or difficulties receiving appropriate support, particularly where services were difficult to navigate or referral routes were unclear (Healthwatch Southwark, 2023; Montañez, 2020; Transformation Partners in Health and Care, 2024).

Several publications suggested that services were not always perceived as responsive to Latin American communities. Participants described limited provider understanding of migration histories, social circumstances and cultural contexts shaping distress and help-seeking, alongside interactions experienced as impersonal, dismissive or lacking warmth (Guerra et al., 2022; Healthwatch Islington, 2016; Healthwatch Southwark, 2023; Lopez et al., 2014; Montañez, 2020). These barriers appeared to affect both initial access to support and continued engagement with care.

#### 3.3.2 Linguistic and cultural inaccessibility

Language barriers emerged as one of the most consistently reported obstacles to accessing mental health support. Publications described limited availability of services delivered in Spanish or Portuguese, lack of translated information and difficulties understanding referral pathways, appointments and written communications (Healthwatch Islington, 2016; Healthwatch Southwark, 2023; Lopez et al., 2014; McIlwaine and Carlisle, 2011; Montañez, 2020; Southwark Council, 2024).

Although interpreter services were sometimes available, participants described additional waiting times, restricted opportunities to explain their concerns fully and uncertainty about the accuracy of interpretation, particularly when relying on family members or friends (Healthwatch Islington, 2016). Beyond language, participants described statutory services as culturally distant or insufficiently responsive to Latin American communities, while shared language, cultural familiarity, warmth and trust were identified as important for engagement (Cordoba et al., 2025; Ghobrial et al., 2025; Guerra et al., 2022; Healthwatch Southwark, 2023; Lopez et al., 2014; Southwark Council, 2024; Transformation Partners in Health and Care, 2024).

#### 3.3.3 Immigration insecurity as a barrier

Immigration-related concerns played a significant role in shaping help-seeking. Fear of immigration enforcement, NHS charging and possible data-sharing with the Home Office discouraged some individuals, particularly those with insecure or uncertain immigration status, from seeking healthcare (Coalition of Latin American Organisations in the UK, Latin American Women’s Rights Service, 2020; Healthwatch Southwark, 2023; McIlwaine and Carlisle, 2011). NRPF restrictions were also described as limiting access to housing, welfare support and financial stability, increasing vulnerability and reducing capacity to seek help (Cordoba et al., 2025; McIlwaine and Carlisle, 2011; National AIDS Trust, 2021).

Practical barriers associated with precarious employment and socio-economic insecurity were also commonly reported. Long working hours, insecure contracts, unstable housing and fear of income loss made it difficult to prioritise or attend appointments, particularly among migrants working in low-paid sectors (Coalition of Latin American Organisations in the UK, Latin American Women’s Rights Service, 2020; de la Silva et al., 2019; McIlwaine and Carlisle, 2011). Across the literature, mental health support was often deprioritised relative to more immediate concerns such as housing, employment, immigration advice and financial security.

#### 3.3.4 Community organisations as gateways to support

In contrast to barriers within formal services, community organisations were frequently described as trusted gateways to support. Organisations including LAWRS, IRMO and CLAUK provided translation, advocacy, navigation support, practical assistance, emotional support and, in some cases, referral pathways into formal mental health services or direct counselling and peer-based support (Berg, 2017; Coalition of Latin American Organisations in the UK, Latin American Women’s Rights Service, 2020; de la Silva et al., 2019; Evans and McIlwaine, 2017; Healthwatch Southwark, 2023; McIlwaine and Carlisle, 2011; Montañez, 2020; National AIDS Trust, 2021).

Community organisations were often described as culturally familiar and trusted spaces where individuals felt understood and respected, particularly where trust in statutory services was limited (Guerra et al., 2022; McIlwaine et al., 2011, Montañez, 2020). These organisations appeared to function as intermediaries between individuals and wider systems of care, supporting disclosure, service navigation and access to specialist support (Healthwatch Southwark, 2023; McIlwaine and Carlisle, 2011).

#### 3.3.5 Informal support pathways

Alongside community organisations, informal support networks played an important role in supporting wellbeing and coping with distress. Peer relationships, family members, transnational family networks, faith communities and online groups provided emotional support, guidance, practical assistance, belonging and information about services (Caicedo, 2023, Guerra et al., 2022, Healthwatch Southwark, 2023, McIlwaine et al., 2011, Montañez, 2020, te Lintelo et al., 2024, Turcatti and Vargas-Silva, 2021). For some participants, particularly sexual and gender minority individuals, chosen family networks were important where biological family relationships were distant or strained (Cordoba et al., 2025; Ghobrial et al., 2025). Research involving young people from racially minoritised backgrounds, including Latin Americans, also highlighted the importance of trusted adults, youth workers and community-based youth groups (Transformation Partners in Health and Care, 2024).

Across the literature, informal and community-based support networks appeared to complement rather than replace formal mental health care, helping individuals cope with distress and navigate wider pathways to support.

## 4 Discussion

### 4.1 Mental health and wellbeing in the context of migration and settlement

This scoping review found that the available literature primarily emphasised the social, structural and relational conditions associated with migration and settlement, rather than specific diagnostic profiles. Distress was linked to immigration insecurity, labour and housing precarity, family separation, discrimination and barriers to healthcare, while wellbeing was associated with social connection, cultural continuity and trusted community spaces.

These findings align with migration-health literature showing that mental health outcomes are shaped by the structural and social conditions associated with migration and settlement rather than migration status alone (Bhugra, 2004; Hynie, 2018). They also resonate with international literature on Latino, Hispanic and Latin American-origin migrant mental health. Studies from the United States, Spain and other European contexts have linked psychological distress to migration stressors, documentation status, discrimination and post-migration insecurity (Alarcão et al., 2023, Alegría et al., 2008; Fortuna et al., 2016; Garcini et al., 2021). The emphasis on labour precarity and workplace discrimination in this review is also consistent with evidence from European migrant populations that discrimination at work can adversely affect mental health (Di Napoli et al., 2021). Findings relating to family, social connection and culturally shaped help-seeking similarly reflect wider literature on familism, stigma, language and mental health service use among Latino populations (Derr, 2016; Grieb et al., 2023; Teruya and Bazargan-Hejazi, 2013; Valdivieso-Mora et al., 2016; Villatoro et al., 2014). These parallels suggest that many of the stressors identified in this review are not unique to the UK, but reflect broader dynamics affecting Latin American-origin migrants across different destination contexts. However, this review sharpens understanding of how these dynamics are configured in the UK through institutional invisibility, NHS access routes, immigration policy, welfare eligibility, post-Brexit insecurity and reliance on community-sector support.

Institutional invisibility may also shape mental health and access to care. Although many barriers identified in this review mirror those experienced by other migrant and racially minoritised communities, the absence of a distinct Latin American category within national ethnic monitoring systems may obscure needs within broader residual categories. This may limit recognition within routine health data, commissioning and service planning, making unmet need harder to identify and inequalities easier to overlook.

The findings should also be interpreted in light of the heterogeneity contained within the category “Latin American”. Umbrella categories can improve visibility and help identify shared patterns of marginalisation, but may also obscure differences by national origin, language, racialised identity, migration pathway, generation, legal status and socio-economic position (Aragones et al., 2014; Errisuriz et al., 2024). Themes such as language barriers, labour precarity, immigration-related insecurity and reliance on community organisations should therefore not be read as uniform Latin American experiences, but as patterns within a heterogeneous evidence base spanning different populations and contexts.

### 4.2 Accessing mental health support: pathways, candidacy and distributed systems of care

Access to mental health support was best understood as a pathway rather than a single point of entry. Difficulties emerged across recognising distress, deciding whether support was needed, navigating systems and sustaining engagement. This supports wider UK literature showing that ethnic inequalities in mental healthcare arise across the pathway to care, not only at service entry (Alam et al., 2024; Bansal et al., 2022; Harwood et al., 2023; Lowther-Payne et al., 2023).

Difficulties accessing formal care appeared to arise through an interaction between general system pressures and migration-related barriers, including long waiting times, limited awareness of services, uncertainty about entitlement, fears relating to immigration status or charging, language barriers and concerns about culturally responsive care. These experiences mirror barriers reported across other racially minoritised communities in the UK, including mistrust, stigma, previous negative encounters and fears of discrimination (Alam et al., 2024; Bansal et al., 2022). The concept of candidacy is useful here because it shows how eligibility for healthcare is jointly negotiated between individuals and services, rather than determined only by whether a service formally exists (Dixon-Woods et al., 2006). For some Latin Americans, candidacy may be further constrained by low visibility within services, limited tailoring of provision and institutional mistrust linked to legal precarity or documentation status (Derr, 2016; Garcini et al., 2021; Martinez et al., 2015).

In the UK context, barriers to care were linked not only to individual help-seeking but also to NHS navigation, uncertainty around entitlement and charging, NRPF-related welfare and housing restrictions and post-Brexit insecurity affecting some onward Latin American migrants from Europe. Recognising distress did not necessarily translate into help-seeking unless individuals had knowledge of therapy, familiarity with mental health systems or contact with trusted people and organisations who could support navigation. Inequalities may therefore begin before formal service contact.

Language emerged as both an access issue and a therapeutic issue. The concern was not only whether people could understand information or appointments, but whether sensitive psychological experiences could be communicated with emotional nuance, confidentiality and relational safety, particularly when interpreters were involved (Krystallidou et al., 2024; Tribe and Thompson, 2026). Community organisations and informal networks also appeared to function as access infrastructure rather than peripheral support. By providing advocacy, translation, navigation, practical assistance and emotional safety, community organisations, churches and local community centres helped bridge the gap between individuals and formal systems, particularly where people were unfamiliar with services, uncertain about eligibility or mistrustful of statutory provision. This reinforces the value of understanding help-seeking as a process shaped by recognition, intention, access routes and available support, rather than as a single service-use event (McLaren et al., 2023).

### 4.3 Implications for practice, policy and future research

These findings suggest that improving access may involve culturally responsive and language-concordant care, clearer referral pathways and stronger partnerships with trusted community organisations. Clinically, greater attention to migration experiences and structural inequalities may support assessment and engagement, while community organisations may facilitate outreach and service navigation. At a policy level, improved recognition of Latin Americans within health data and commissioning, alongside consideration of wider immigration, welfare and housing policies, may help address unmet needs.

Future research should examine how mental health needs and pathways differ across national origin groups, languages, racialised identities, migration pathways, generations and immigration statuses, including among Latin American communities outside of London. Research should also incorporate the perspectives of both Latin American service users and clinicians and community practitioners, including those of Latin American heritage, to better understand how cultural familiarity, language and trust shape experiences of care. Longitudinal research could further explore how help-seeking pathways develop over time, while evaluations of culturally adapted interventions and community-based approaches could help identify effective strategies for improving access and engagement.

### 4.4 Strengths and limitations

This review synthesised evidence from both peer-reviewed and grey literature, allowing inclusion of community knowledge often absent from academic databases. This was particularly valuable given the scarcity of peer-reviewed UK research focused specifically on Latin Americans and the importance of community organisations, advocacy groups and local services in producing relevant evidence.

Several limitations should be acknowledged. The evidence base was heavily concentrated in London, reflecting the substantial proportion of the UK's Latin American population residing in the capital, although this may limit the transferability of findings to other UK settings. Publications varied in methodology, sample size and population characteristics, requiring caution when interpreting findings across different contexts and subgroups. Thematic coding was conducted by a single reviewer, meaning their perspective may have influenced the interpretation and development of themes, although these were reviewed and refined through supervision and wider team discussion. The category “Latin American” is highly heterogeneous and contested; although used pragmatically in this review, the term may obscure differences by national origin, language, racialised identity, migration pathway, generation, legal status and socio-economic position (Aragones et al., 2014; Errisuriz et al., 2024).

The grey literature search also has limitations. Google Advanced Search can be difficult to reproduce because results vary over time and by search settings, location and indexing changes. Structured grey literature databases were not searched, which may have resulted in indexed reports, theses or policy documents being missed. However, transparency was strengthened by recording search terms, dates, URLs and screening decisions and by supplementing searches with targeted organisational website searches and citation tracking. Grey literature varied in methodological transparency, although included reports were required to present primary empirical data and many were produced by credible organisations with community expertise. The review was not pre-registered. Searching was limited to PsycINFO, MEDLINE and EMBASE, so relevant literature, particularly from Latin American-focused databases such as LILACS and SciELO, may have been missed.

### 4.5 Conclusion

The reviewed literature linked mental health and wellbeing among Latin Americans in the UK to migration-related stressors, socio-economic precarity and discrimination alongside sources of resilience rooted in family, community and cultural continuity. Institutional invisibility emerged as an additional challenge for recognising needs and providing accessible support. Help-seeking involved formal, informal, community-based and transnational systems rather than through a single linear route. While these findings are consistent with international literature on Latin American migrant mental health, this review highlights how broader migration-related challenges are influenced by NHS access, UK immigration and welfare policies and reliance on community support. Improving mental health equity may therefore require culturally responsive care, stronger partnerships with community organisations, greater visibility within health systems and attention to the wider social conditions shaping wellbeing and access.

## Funding

The authors received no specific funding for this work.

## Data availability statement

This is a scoping review; all data are drawn from published documents cited in the manuscript.

## CRediT authorship contribution statement

**Rhysha Gallo:** Writing – review & editing, Writing – original draft, Visualization, Project administration, Methodology, Investigation, Formal analysis, Data curation, Conceptualization. **María Camila García Durán:** Writing – review & editing, Validation, Investigation. **Vaughan Bell:** Writing – review & editing, Supervision, Methodology, Conceptualization. **Norha Vera San Juan:** Writing – review & editing, Supervision, Methodology, Conceptualization.

## Declaration of competing interest

The authors declare no conflicts of interest.
