# Supplementary material for: Mental health and access to support among Latin Americans in the United Kingdom: A scoping review
Source: J Migr Health. 2026 Oct 29;14:100457. doi: 10.1016/j.jmh.2026.100457 (PMC13757499; doi:10.1016/j.jmh.2026.100457)
Supplement: Supplementary file 1 [file mmc1.docx]

Supplementary Material for:

**Mental Health and Access to Mental Health Support Among Latin Americans in the United Kingdom: A Scoping Review**

**Appendix A.** **Full Search Strategy**

This appendix outlines the search strategy used for academic database and grey literature searches. Search terms were adapted across databases according to indexing requirements and search functionality.

*Academic Database Search Strategy*

Searches were conducted across PsycINFO, MEDLINE and EMBASE using combinations of population, geographic and mental health/support access terms.

*Category 1: Population Terms (Latin American Communities)*

Latin American OR Latinx OR Latino OR Hispanic OR Mexican OR Central American OR South American OR Brazilian OR Colombian OR Ecuadorian OR Peruvian OR Chilean OR Bolivian OR Argentinian OR Venezuelan OR migrant OR immigrant

*Category 2: Geographic Terms (UK Context)*

UK OR Britain OR British OR England OR Scotland OR Wales OR Northern Ireland OR London OR “British context” OR “in the UK”

*Category 3: Mental Health, Wellbeing and Service Access Terms*

Mental health OR mental illness OR mental disorder OR psychiatric disorder OR psychological distress OR emotional distress OR emotional wellbeing OR depression OR anxiety OR PTSD OR post-traumatic stress OR suicid OR low mood OR wellbeing OR help seeking OR support seeking OR healthcare access OR mental health service OR access to mental health OR barriers to care OR service use OR utilisation OR informal support OR professional help OR pathway to care OR referral OR outreach

Search terms across categories were combined using Boolean operators (AND/OR).

*Grey Literature Search Terms*

Grey literature searches were conducted using Google Advanced Search and targeted searches of relevant organisational websites.

The following search string was used in Google Advanced Search, with results restricted to the United Kingdom using the geographic filtering function:

"Latin American" AND ("mental health" OR "wellbeing" OR "access") AND ("UK" OR "England" OR "Wales" OR "Scotland" OR "Northern Ireland")

Backward and forward citation tracking of included publications and relevant reports was also undertaken.

**Appendix B. Eligibility Criteria**

The following inclusion and exclusion criteria were applied during title/abstract and full-text screening.

| *Domain* | *Inclusion Criteria* | *Exclusion Criteria* |
| --- | --- | --- |
| Population | Studies including Latin Americans living in the UK | Studies not including Latin Americans or where findings relating to Latin Americans could not be distinguished from broader ethnic groups |
| Context | Research conducted within the United Kingdom | Research conducted outside the UK |
| Topic | Studies examining mental health needs, psychological wellbeing and/or experiences of accessing or seeking formal or informal mental health support | Studies not relating to mental health, wellbeing or support access |
| Study Design | Qualitative, quantitative and mixed-methods studies | Non-empirical publications |
| Source Type | Peer-reviewed and grey literature reporting primary empirical data | Opinion pieces, editorials, commentaries and publications not reporting primary empirical data |
| Language | No language restrictions applied | Not applicable |
| Publication Date | No publication date restrictions applied | Not applicable |

**Table S1.** Eligibility Criteria Used During Screening

**Appendix C. PRISMA-ScR Checklist**

| *Section* | *Item* | *Prisma-SCR Checklist Item* | *Reported in Manuscript* |
| --- | --- | --- | --- |
| Title | | | |
| Title | 1 | Identify the report as a scoping review. | Title: Mental Health and Access to Support Among Latin Americans in the United Kingdom: A Scoping Review |
| Abstract | | | |
| Structured Summary | 2 | Provide a structured summary that includes (as applicable): background, objectives, eligibility criteria, sources of evidence, charting methods, results, and conclusions that relate to the review questions and objectives. | Abstract: Background; Methods; Results; Conclusions |
| Introduction | | | |
| Rationale | 3 | Describe the rationale for the review in the context of what is already known. Explain why the review questions/objectives lend themselves to a scoping review approach. | Background and Context |
| Objectives | 4 | Provide an explicit statement of the questions and objectives being addressed with reference to their key elements. | Objective |
| Methods | | | |
| Protocol and Registration | 5 | Indicate whether a review protocol exists; state if and where it can be accessed; and if available, provide registration information. | Protocol not registered |
| Eligibility Criteria | 6 | Specify characteristics of the sources of evidence used as eligibility criteria and provide a rationale. | Methods/Inclusion Criteria; Appendix B. Eligibility Criteria |
| Information Sources | 7 | Describe all information sources in the search, as well as the date the most recent search was executed. | Methods/Search Strategy |
| Search | 8 | Present the full electronic search strategy for at least one database, including any limits used. | Appendix A. Full Search Strategy |
| Selection of Sources of Evidence | 9 | State the process for selecting sources of evidence included in the scoping review. | Methods/Source Selection; Figure 1; Appendix F. Full-Text Exclusions with Reasons |
| Data Charting Process | 10 | Describe the methods of charting data from the included sources of evidence. | Methods/Data Extraction and Synthesis; Appendix D. Data Extraction Framework |
| Data Items | 11 | List and define all variables for which data were sought and any assumptions and simplifications made. | Methods/Data Extraction and Synthesis; Appendix D. Data Extraction Framework |
| Critical Appraisal of Individual Sources of Evidence | 12 | If done, provide a rationale for conducting a critical appraisal of included sources of evidence; describe the methods used and how this information was used in any data synthesis. | Methods/Data Extraction and Synthesis; Appendix E. AACODS Quality Appraisal (Grey Literature) |
| Synthesis of Results | 13 | Describe the methods of handling and summarizing the data that were charted. | Methods/Data Extraction and Synthesis |
| Results | | | |
| Selection of Sources of Evidence | 14 | Give numbers of sources of evidence screened, assessed for eligibility and included in the review, with reasons for exclusions at each stage, ideally using a flow diagram. | Methods/Source Selection; Figure 1; Appendix F. Full-Text Exclusions with Reasons |
| Characteristics of Sources of Evidence | 15 | For each source of evidence, present characteristics for which data were charted and provide the citations. | Results/Literature Characteristics; Table 1; Appendix D. Data Extraction Framework |
| Critical Appraisal Within Sources of Evidence | 16 | If done, present data on critical appraisal of included sources of evidence. | Appendix E. AACODS Quality Appraisal of Grey Literature Sources |
| Results of Individual Sources of Evidence | 17 | For each included source of evidence, present the relevant data that were charted that relate to the review questions and objectives. | Results/Mental Health Needs and Psychological Wellbeing; Results/Experiences of Accessing Mental Health Support; Appendix D. Data Extraction Framework |
| Synthesis of Results | 18 | Summarize and/or present the charting results as they relate to the review questions and objectives. | Results |
| Discussion | | | |
| Summary of Evidence | 19 | Summarize the main results, link to the review questions and objectives, and consider the relevance to key groups. | Discussion |
| Limitations | 20 | Discuss the limitations of the scoping review process. | Strengths and Limitations |
| Conclusions | 21 | Provide a general interpretation of the results with respect to the review questions and objectives, as well as potential implications and/or next steps. | Conclusion; Future Research |
| Funding | | | |
| Funding | 22 | Describe sources of funding for the included sources of evidence, as well as sources of funding for the scoping review. | Title Page/Funding |

From: Tricco AC, Lillie E, Zarin W, et al. PRISMA extension for scoping reviews (PRISMA-ScR): Checklist and explanation. Ann Intern Med. 2018;169(7):467-473. doi:10.7326/M18-0850

**Appendix D. Data Extraction Framework**

This table presents the full descriptive charting framework used to extract study information, methodological characteristics, population details, findings relating to mental health and support access, and AACODS appraisal domains for grey literature sources.

| *Main Heading* | *Data Extracted* |
| --- | --- |
| Source Information | Title; author; academic/grey literature; publication year; organisation; location of the team; journal; reliability note |
| Study Design and Methods | Study type: qualitative, quantitative or mixed methods; design/method; recruitment; main research questions and aims |
| Population Description | Location of the study; population characteristics, including age, gender, migration status, ethnicities and languages; age; whether the publication was Latin American-specific; whether it focused on a specific Latin American ethnic/national group |
| Mental Health Needs and/or Psychological Wellbeing | Whether mental health needs/psychological wellbeing were discussed; measures used; main findings/themes; notes |
| Experiences of Accessing Mental Health Support | Whether experiences of accessing mental health support were discussed; whether informal support was included; measures used; main findings/themes; notes |
| Grey Literature Quality Appraisal (AACODS) | Authority; accuracy; objectivity |

**Table S2.** Descriptive Charting and Data Extraction Framework

**Note.** Academic/grey literature refers to whether the source was a peer-reviewed academic publication or a grey literature source. AACODS = Authority, Accuracy, Coverage, Objectivity, Date, Significance; selected AACODS domains used in this review were authority, accuracy and objectivity.

This appendix summarises the thematic charting framework used to organise and synthesise findings across included publications. The framework was operationalised within the thematic extraction spreadsheet used during the synthesis process and applied iteratively across both analytic domains: (1) mental health needs and psychological wellbeing and (2) experiences of accessing mental health support.

| *Main Heading* | *Data Extracted* |
| --- | --- |
| Main Theme | Broad thematic category identified across included publications |
| Subtheme | More specific thematic concepts grouped within each broader theme |
| Studies | Publications contributing evidence to each subtheme  Publications contributing to each subtheme were organised according to the extent to which themes were emphasised within the source material:  Strong evidence: the subtheme was explicitly discussed and central to the publication findings  Moderate evidence: the subtheme was clearly present but not always a primary focus or discussed in depth  Contextual/Indirect evidence: the subtheme was referenced more indirectly or discussed as broader contextual information rather than analysed as a standalone finding  These categories were used descriptively to support transparency in thematic synthesis rather than to formally assess methodological quality or weight of evidence |
| Summary Finding | Concise summary of the main findings relating to the subtheme across included publications |
| Population Notes | Relevant population-specific observations, including differences relating to gender, migration status, sexuality, nationality or other contextual factors |
| Interpretation/Relevance | Brief notes regarding the relevance of the subtheme to the review aims and wider understanding of mental health and wellbeing among Latin Americans in the UK |

**Table S3.** Thematic Charting Framework Used Across Both Analytic Domains

**Appendix E. AACODS Quality Appraisal of Grey Literature Sources**

Grey literature sources were appraised using selected AACODS domains focusing on authority, accuracy and objectivity. Appraisal aimed to support transparency regarding the credibility and methodological quality of included grey literature sources rather than to exclude sources on the basis of quality.

| *Publication* | *Authority* | *Accuracy* | *Objectivity* |
| --- | --- | --- | --- |
| HIV and Migration: Understanding the Barriers Faced by People Born Abroad Living With HIV in the UK | Produced by National AIDS Trust (national policy organisation). Recognised expertise in HIV policy. Advisory group included individuals with lived experience, clinicians, academics and statutory partners. | Not peer-reviewed. Some methodological transparency. Analytic procedures less detailed. Acknowledgement of limitations. | Advocacy-oriented research with policy focus. Findings supported by qualitative data and demographic reporting. |
| Access to Health and Social Care Services for Latin American Communities in Southwark | Statutory Healthwatch body (Healthwatch Southwark), part of national Healthwatch network under Health and Social Care Act 2012. Credible local public health authority. | Clear methodology (survey n=38; interviews n=15; focus group n=14; total n=67). Data collection period specified (Dec 2022–June 2023). Inductive thematic analysis described. Limitations acknowledged. Supported by participant quotes and descriptive statistics. | Community-focused and equity-driven but findings are data-led and transparently reported. Recommendations grounded in participant data. |
| Latin American Health: Southwark’s Joint Strategic Needs Assessment | Produced by Southwark Public Health Division as part of the statutory Joint Strategic Needs Assessment (JSNA) process. Named author and contributors listed. Institutional oversight and NHS Integrated Care Board involvement. High institutional credibility. | Report is referenced and draws on official datasets (Census, NHS dashboards) and published literature. However, primary qualitative engagement methods lack detail (no sampling strategy, no demographic breakdown, no analytic framework described), limiting transparency of the primary data component. | Policy-oriented and needs-based framing. Balanced tone focused on identifying inequalities and service gaps. No overt advocacy bias, though some sections draw on reports from community organisations. |
| Written Evidence Submitted by the Coalition of Latin American Organisations in the UK (CLAUK) and Latin American Women’s Rights Service (LAWRS) | Produced by CLAUK (coalition of 13 Latin American ‘led-by and for’ organisations) and LAWRS, established specialist migrant women’s rights organisation. Submitted to UK Parliament Women & Equalities Committee. Strong community authority; not academic authorship. | Draws on frontline casework evidence and an IRMO survey (100+ beneficiaries) but methods, sampling and analytic processes are not fully described. Includes references to published research. Practice-based evidence rather than systematically collected research. | Advocacy-oriented submission aiming to influence policy. Emphasises structural inequalities and service gaps. Tone is persuasive but evidence-based; may foreground negative experiences due to remit. |
| Listening to Children and Young People to Improve Equity in Mental Health Support for Racially Minoritised Communities in London | Produced by Transformation Partners in Health and Care (TPHC) in partnership with NHS England and London mental health trusts. Authors include senior public health and mental health professionals. Credible institutional backing. | Based on primary qualitative engagement (focus groups, workshops, discussions) with young people from racially minoritised communities. Direct quotes included. However, limited methodological detail (sampling, recruitment, analysis transparency). Not peer-reviewed. | Advocates for anti-racist and equity-focused reform. Normative stance is explicit but grounded in participant accounts. Some emphasis on policy recommendations beyond data. |
| Lemme Hablar: Community Engagement and the Latin American Community in Southwark | Mental health service access is discussed explicitly but embedded within broader service-access and engagement themes. Informal/community-based routes are central to support pathways. No detailed individual service-use trajectories provided. | Report authored by a community practitioner with direct engagement in Latin American organisations (e.g. VOADES, LAWRS, IRMO). Produced within a recognised community engagement context. Not peer-reviewed but grounded in direct fieldwork and practitioner expertise. | Advocacy-oriented and strengths-based. Aims to highlight structural barriers and promote culturally appropriate services. Perspective is community-centred rather than neutral/academic but arguments are transparent and consistent with cited evidence. |
| Latin Americans in Southwark: Service Access and Provision | Authored by Dr Mette Louise Berg (COMPAS, University of Oxford). Conducted as part of an ESRC-funded Knowledge Exchange Fellowship in collaboration with Southwark Council and LAWRS. Strong institutional credibility (University of Oxford + local authority partnership). | Based on qualitative interviews/workshops with service providers and supported by commissioned 2011 Census analysis. Methods are clearly described. However, no direct migrant primary data and no demographic breakdown of interviewees. Not peer-reviewed. | Analytical and policy-oriented. Aims to identify barriers and improve service provision. Some reliance on service-provider perspectives may introduce professional framing bias. Balanced tone; acknowledges both strengths and vulnerabilities of the community. |
| Access to Primary Care and Social Care Services in Islington for Latin Americans Over 50s | Produced by Healthwatch Islington, based on research conducted by LAWRS and CLAUK, established third-sector organisations working with Latin American migrants in London. Healthwatch is part of a statutory national network. Strong community and institutional credibility, though not academic authorship. | Primary data collected (22 participants) via focus groups, interviews and phone surveys. Methods described clearly and demographic breakdown provided. However: small sample size; no validated measures; no peer review; limited methodological detail (e.g. recruitment process, data analysis procedures). Reliable for descriptive community insight but not generalisable. | Policy-oriented and advocacy-informed, aiming to improve service provision. Includes direct participant quotes (strength). Some framing influenced by voluntary-sector perspective but findings appear balanced and evidence-based rather than ideological. |
| The Experiences of London’s Latin American Migrants During Brexit and the COVID-19 Pandemic: An Exploratory Qualitative Study | Produced by Domiziana Turcatti and Carlos Vargas-Silva at the Centre on Migration, Policy and Society (COMPAS), University of Oxford, in collaboration with Latin American House. COMPAS is a recognised academic research centre specialising in migration research, giving the report strong institutional credibility despite being grey literature. | Primary qualitative data collected through focus groups and interviews with 73 Latin American migrants, 15 Latin American students and 39 NGO/community workers. Methods and participant demographics are described and direct quotations from participants support findings. However, the report is an executive summary, with limited methodological detail and no peer review. | The report aims to document community experiences and inform policy responses. Findings are presented through participant testimonies and community perspectives. While produced in collaboration with a community organisation, the report appears balanced and evidence-based rather than overtly advocacy-driven. |
| The Unheard Workforce: Experiences of Latin American Migrant Women in Cleaning, Hospitality and Domestic Work | Produced by the Latin American Women’s Rights Service (LAWRS), a UK-based specialist NGO supporting Latin American migrant women. Authors are researchers and practitioners within the organisation. While not peer-reviewed, the organisation is recognised in research and policy work on migrant rights and violence against women in the UK. | The report draws on 326 anonymised case records from LAWRS’ Employment Rights Advice Service (2015–2018) and survey responses from 52 domestic workers. Methods and sample characteristics are described, and findings are supported with descriptive statistics and case examples. However, the methodology is not highly detailed and the analysis is descriptive, with no formal academic validation. | The report has a clear advocacy orientation, aiming to highlight exploitation and improve labour protections for migrant women. While the report is evidence-based, its framing is aligned with human rights and policy advocacy goals, which may influence emphasis and interpretation. |
| Towards Visibility: The Latin American Community in London | Produced by Cathy McIlwaine and colleagues at Queen Mary University of London in collaboration with the Latin American Women’s Rights Service (LAWRS). The authors are established researchers on Latin American migration in the UK. | Based on primary mixed-methods research including a survey of 400 Latin American migrants and qualitative interviews, alongside analysis of secondary datasets (e.g. Census). Methods and data sources are clearly described, increasing reliability. | The report aims to increase visibility and understanding of the Latin American community in London. While produced partly with community organisations, the research uses systematic data collection and presents findings in a balanced analytical manner. |
| No Longer Invisible: The Latin American Community in London | The report was produced by researchers at Queen Mary University of London in collaboration with Latin American Women’s Rights Service. The lead author, Cathy McIlwaine, is an established academic in migration and urban studies. | The report uses a mixed-methods design including surveys (n=453 and n=509), interviews, focus groups and secondary data analysis. Methods and sampling are clearly described in the methodology appendix, supporting transparency and reliability. | Although the report aims to inform policy and support the Latin American community, it presents empirical findings based on primary data and includes both quantitative and qualitative evidence. The analysis is evidence-based rather than purely advocacy-driven. |
| The Right to Healthcare: A Community-Led Approach to Better Health Outcomes for the Latin American Community | Produced by the Latin American Women’s Rights Service (LAWRS) and Indoamerican Refugee and Migrant Organisation (IRMO), established community organisations supporting Latin American migrants in the UK. The report was written by Carolina Caicedo and funded by Southwark Council, indicating institutional backing and subject expertise in migrant health and rights. | Evidence is drawn from primary project data, including outreach engagement with 657 community members, in-depth support for 144 individuals and 7 qualitative interviews with beneficiaries. The report includes descriptive statistics and case studies, though methods are not fully detailed and findings are not peer-reviewed. | The report aims to highlight healthcare inequalities and advocate for improved access for Latin American migrants. While it presents evidence from outreach data and lived experiences, it has a clear advocacy perspective, reflecting the organisations’ mission to support migrant communities. |
| Supporting Brazilian Women in London: Service Provision for Survivors of Violence Against Women and Girls (VAWG) | Produced by the Latin American Women’s Rights Service (LAWRS) with academic collaboration from Cathy McIlwaine (Queen Mary University of London), a recognised researcher on Latin American migration in the UK. Authors have expertise in migrant women’s rights and VAWG research. | The report clearly outlines its research methods (qualitative interviews and stakeholder engagement) and situates findings within existing research on migrant women and VAWG. Evidence is supported with participant quotes and references to prior literature. | While the report has an advocacy focus (aimed at improving service provision), findings are grounded in participant narratives and service provider perspectives, with transparent discussion of barriers faced by migrant women. |

**Table S4.** AACODS Appraisal of Included Grey Literature Sources

**Note.** AACODS = Authority, Accuracy, Coverage, Objectivity, Date, Significance. Consistent with the review aims, selected AACODS domains relating to authority, accuracy and objectivity were applied. Full extraction details are provided in Appendix D.

**Appendix F. Full-Text Exclusions with Reasons (n=465)**

The following publications were excluded following full-text eligibility assessment. Publications are grouped by standardised exclusion criteria and listed using short citations generated from the author, year and title fields. Exclusion reasons were standardised from screening notes to align with the review eligibility criteria.

*Does not include/Unclear if they included Latin Americans living in the UK (n=232)*

- Aalto et al. (2025). Patterns of mental health problems and resilience among immigrant and refugee adolescents: A latent profile analysis.
- Abdel-Salam (2014). Examining the relationship between self-control and adolescent TC treatment completion.
- Agbonmwandolor et al. (2024). Coping strategies used by migrant healthcare workers to support their mental health during COVID-19 in the United Kingdom: A qualitative analysis.
- Alegria et al. (2008). How missing information in diagnosis can lead to disparities in the clinical encounter.
- Allport et al. (2023). Developmental referrals of pre-school children in a diverse community in England: The importance of parental migration for referral rates.
- Almond (2011). Inequity in provision of and access to health visiting postnatal depression services.
- Anderson et al. (2019). Impact of Insecure Immigration Status and Ethnicity on Antenatal Mental Health Among Migrant Women.
- Anderson et al. (2021). Do outcomes of psychiatric hospital treatment differ for migrants and non-migrants?
- Anglin et al. (2013). Immigrant status, racial discrimination, and clinically high risk for psychosis among racial and ethnic minority youth.
- Aronson and Bergh (2021). "Adolescents who feel depressed are rejected but do not withdraw: A longitudinal study of ethnically diverse friendship networks in England, Sweden, and Germany".
- Astell-Burt et al. (2012). Racism, ethnic density and psychological well-being through adolescence: Evidence from the Determinants of Adolescent Social Well-being and Health longitudinal study.
- Bagley (1971). Mental illness in immigrant minorities in London.
- Bains and Gutman (2021). Mental Health in Ethnic Minority Populations in the UK: Developmental Trajectories from Early Childhood to Mid Adolescence.
- Banerjee et al. (2024). Ethnic diversity of children and young people with attention deficit hyperactivity disorder: Observation from a single centre.
- Bean et al. (2007). Comparing psychological distress, traumatic stress reactions, and experiences of unaccompanied refugee minors with experiences of adolescents accompanied by parents.
- Bhavsar et al. (2021). The association of migration and ethnicity with use of the Improving Access to Psychological Treatment (IAPT) programme: A general population cohort study.
- Blanchflower and Oswald (2008). Is well-being U-shaped over the life cycle?
- Blank et al. (2022). Improvement in Depression and Fluid Cognition Following Accelerated Theta Burst Stimulation for Treatment- Resistant Major Depressive Disorder in Autism Spectrum Disorders.
- Bourque et al. (2012). Family history of mental illnesses in first-and second-generation migrants: Evidence from the AESOP first-onset study
- Bowe (2017). The immigrant paradox on internalizing symptoms among immigrant adolescents.
- Braham et al. (2020). 'I needed somebody like her in my life': Migrant women's experiences and perceptions of health navigation services to increase access to maternity care in the uk.
- Brough et al. (2023). Addressing internalised stigma in HIV clinics: a London Fast-Track Cities Initiative (FTCI) project.
- Burvill et al. (1973). Methods of suicide of English and Welsh immigrants in Australia.
- Campbell et al. (2018). Social determinants of emotional well-being in new refugees in the UK.
- Cardemil et al. (2014). Depressive symptoms in low-income, urban adolescents: Cognitive and contextual factors.
- Carranza and Parshall (2005). Psychiatric morbidity of overseas patients in inner London: A hospital based study.
- Castaneda-Gameros et al. (2018). Physical Activity, Sedentary Time, and Frailty in Older Migrant Women From Ethnically Diverse Backgrounds: A Mixed-Methods Study.
- Chartonas and Bose (2015). Fighting with Spirits: Migration Trauma, Acculturative Stress, and New Sibling Transition-A Clinical Case Study of an 8-Year-Old Girl with Absence Epilepsy.
- Chilvers and Hosie (2015). 'Midwives should not act as gatekeepers to the maternity services'.
- Clark et al. (2022). Scarred survivors: Gate keepers and gate openers to healthcare for migrants in vulnerable circumstances.
- Cleland et al. (2012). Community pharmacists' perceptions of barriers to communication with migrants.
- Coca et al. (2023). Factors associated with postpartum depression symptoms among postpartum women in five countries during the COVID-19 pandemic: An online cross-sectional study.
- Cochrane (1977). Mental illness in immigrants to England and Wales: An analysis of mental hospital admissions, 1971.
- Cochrane (1980). Mental illness in England, in Scotland and in Scots living in England.
- Cochrane and Bal (1988). Ethnic density is unrelated to incidence of schizophrenia.
- Cochrane and Bal (1989). Mental hospital admission rates of immigrants to England: A comparison of 1971 and 1981.
- Cochrane and Stopes-Roe (1981). Social class and psychological disorder in natives and immigrants to Britain.
- Cohen (2008). Safe in our hands?: A study of suicide and self-harm in asylum seekers
- Collery and Niedzwiedz (2024). Climate change worry and risk of depression and anxiety: A longitudinal study across 11 European countries.
- Condon and McClean (2017). Maintaining pre-school children's health and wellbeing in the UK: A qualitative study of the views of migrant parents.
- Corscadden et al. (2018). Factors associated with multiple barriers to access to primary care: An international analysis.
- Cross et al. (2008). Comparative assessment of migrant farm worker health in conventional and organic horticultural systems in the United Kingdom.
- De et al. (2023). The Advancing Mental Health Equality Collaborative: Using Quality Improvement to Advance Equality in Mental Health Care.
- De et al. (2024). The Impact of Migrant-sensitive Strategies in the Treatment of Multi-drug Resistant Tuberculosis.
- Dean et al. (1981). First admissions of native-born and immigrants to psychiatric hospitals in South-East England 1976.
- DesMeules et al. (2004). New approaches to immigrant health assessment.
- Dhadda and Greene (2018). 'The Healthy Migrant Effect' for Mental Health in England: Propensity-score Matched Analysis Using the EMPIRIC Survey.
- Dobbin et al. (2023). Medical consequences of "contingency accommodation" for people seeking asylum: Thematic analysis of a survey from professionals working in contingency accommodation.
- Domene and Morley (2022). Stepping into Salsa culture: An experiential account of engaging with a university non-credit dance programme.
- Dotsikas et al. (2025). Exploring the impact of 'hostile environment' policies on psychological distress of ethnic groups in the UK: A differences-in-differences analysis.
- Duncan et al. (1999). Police reports on domestic incidents involving intimate partners: Injuries and medical help-seeking.
- Dura-Vila et al. (2011). Ethnicity, religion and clinical practice: A qualitative study of beliefs and attitudes of psychiatrists in the United Kingdom.
- Dyer (2011). Treatment of man with mental illness at UK detention centre breached human rights.
- Ekezie et al. (2023). Association between ethnicity and migration status with the prevalence of single and multiple long-term conditions in UK healthcare workers.
- Fazel and Stein (2003). Mental health of refugee children: Comparative study.
- Figueiredo et al. (2020). Enhancing patient safety and access to care to populations at risk of exclusion: The example of the Portuguese-speaking migrant community in South London.
- Filby (2019). Meeting the needs of migrant women: A service evaluation of a specialist migrant maternity service within an initial accommodation unit.
- Finlay-Jones and Burvill (1977). The prevalence of minor psychiatric morbidity in the community.
- Finnerty et al. (2018). The health of recent migrants from resource-poor countries.
- Forrester et al. (2008). Parental substance misuse and child welfare: Outcomes for children two years after referral.
- Frissa et al. (2013). Trauma and current symptoms of PTSD in a South East London community.
- Frost (2020). Hostile and harmful: Structural stigma and minority stress explain increased anxiety among migrants living in the United Kingdom after the Brexit referendum.
- Fu et al. (2022). Vulnerable migrants' access to healthcare in the early stages of the COVID-19 pandemic in the UK.
- Gariepy et al. (2015). The impact of pregnancy diagnosis on depression: Does it differ by self-assessment of pregnancy context?
- Gariepy et al. (2017). Pregnancy context and women's health-related quality of life.
- Gatt et al. (2020). Trauma, resilience, and mental health in migrant and non-migrant youth: An international cross-sectional study across six countries.
- Gazard et al. (2015). Challenges in researching migration status, health and health service use: An intersectional analysis of a South London community.
- Gerling and Age Concern England; Commission for Racial Equality (2003). A comparison of the British and German of community care reaction on ethnic minority senior citizens.
- Gerodetti and Foster (2023). Migrant gardeners, health and wellbeing: Exploring complexity and ambivalence from a UK perspective.
- Gillam et al. (1989). Ethnic differences in consultation rates in urban general practice.
- Gkiouleka et al. (2018). Depressive symptoms among migrants and non-migrants in Europe: documenting and explaining inequalities in times of socio-economic instability.
- Glover (1987). 993W: Birthplace not stated or born at sea.
- Glover (1987). First admissions of native-born and immigrant patients to psychiatric hospitals.
- Gogoi et al. (2025). Migrants Living in the United Kingdom and Their Perceptions of Participation in Health Research: A Mixed-Methods Study.
- Gonzalez et al. (2015). International rates of ADHD and substance use disorder symptoms among prison inmates: Individual-data and pooled effects.
- Gonzalez et al. (2015). International rates of SUD and ADHD symptoms among prison inmates: Individual data and pooled effects.
- Goodwin et al. (2018). Taking an intersectional approach to define latent classes of socioeconomic status, ethnicity and migration status for psychiatric epidemiological research.
- Grenfell et al. (2024). East London Project: A participatory mixed-method evaluation on how removing enforcement could affect sex workers' safety, health and access to services in East London.
- Groark et al. (2011). Understanding the experiences and emotional needs of unaccompanied asylum-seeking adolescents in the UK.
- Hameed et al. (2023). Meeting the Needs of Asylum Seekers & Refugees: The Norfolk Experience.
- Harold and Dienes (2019). The influence of ethnicity and nationality on cortisol reactivity to an acute laboratory stressor, with and without partner support.
- Hashmi (1970). Immigrants and emotional stress.
- Hems and Crowe (1999). The psychosexual dysfunction clinic at the Maudsley Hospital, London: A survey of referrals between January and December 1996.
- Hernandez et al. (2010). Ethnic group differences in substance use, depression, peer relationships, and parenting among adolescents receiving brief alcohol counseling.
- Hewetson (1975). Homeless people as an at-risk group.
- Hirsch et al. (2012). Race and ethnic differences in hope and hopelessness as moderators of the association between depressive symptoms and suicidal behavior.
- Hitch and Clegg (1980). Modes of referral of overseas immigrant and native-born first admissions to psychiatric hospital.
- Honaker et al. (2020). Insomnia in adolescents: Patient-centered outcomes and perspectives.
- Humphrey et al. (2025). "I can't show them on the phone so it's what I say and I'm not saying a lot." - The loss of nonverbal and visual cues during telephone consultations, equity of access and the impact on marginalised patients: A qualitative study.
- Ikram et al. (2016). All-cause and cause-specific mortality of different migrant populations in Europe.
- Ineichen et al. (1984). Psychiatric hospital admissions in Bristol. I. Geographical and ethnic factors.
- Isaacs et al. (2022). 'I don't think there's anything I can do which can keep me healthy': How the UK immigration and asylum system shapes the health & wellbeing of refugees and asylum seekers in Scotland.
- Jackson et al. (2012). Nativity differences in mothers' health behaviors: A cross-national and longitudinal lens.
- Jacobs et al. (2001). Impact of interpreter services on delivery of health care to limited-English-proficient patients.
- Jannesari et al. (2022). What affects the mental health of people seeking asylum in the UK? A narrative analysis of migration stories.
- Jayati et al. (2013). Psychosocial adversity in childhood accounts for poorer mental and physical health problems in adulthood in second generation irish people in Britain.
- Jayaweera et al. (2010). Health status, health behaviour and healthcare use among migrants in the UK: Evidence from mothers in the Millennium Cohort Study.
- Jenkins et al. (1981). Psychiatric admissions and aircraft noise from London Airport: Four-year, three-hospitals' study.
- Jidong et al. (2020). Challenges faced by mental health interpreters in East London: An interpretative phenomenological analysis.
- Kanagaratnam and Asbjornsen (2007). Executive deficits in chronic PTSD related to political violence.
- Kellezi et al. (2021). Healthcare provision inside immigration removal centres: A social identity analysis of trust, legitimacy and disengagement.
- Kienast-von et al. (2023). Residential relocation in the UK: Differences in health, behaviour and neighbourhood perceptions between adult newcomers and settled residents.
- Kilpi-Jakonen and Kwon (2023). The Behavioral and Mental Health Benefits of Speaking the Heritage Language within Immigrant Families: The Moderating Role of Family Relations.
- Kirkbride et al. (2016). Do symptom dimensions vary between ethnic groups at first presentation to early intervention in psychosis services? Evidence from the SEPEA study.
- Kirkbride et al. (2017). Ethnic Minority Status, Age-at-Immigration and Psychosis Risk in Rural Environments: Evidence from the SEPEA Study.
- Knipe et al. (2024). Ethnicity and suicide in England and Wales: A national linked cohort study.
- Kraus (1969). The relationship of psychiatric diagnoses, hospital admission rates, and size and age structure of immigrant groups.
- Kryter (1990). Aircraft noise and social factors in psychiatric hospital admission rates: A re-examination of some data.
- Langa et al. (2009). Cognitive health among older adults in the United States and in England.
- Lax et al. (2024). Impact of glucocorticoids on appearance and sense of self in rheumatic diseases.
- Leavey et al. (2004). Psychological disorder amongst refugee and migrant schoolchildren in London.
- Levy (2010). Medication use by ethnic and racial groups: Policy implications.
- Lim (1983). A psychiatric emergency clinic: A study of attendances over six months.
- Lissauer et al. (1993). Influence of homelessness on acute admissions to hospital.
- Littlewood and Lipsedge (1978). Migration, ethnicity and diagnosis.
- Livingston et al. (2001). Mental health of migrant elders--the Islington study.
- Livingston et al. (2002). Accessibility of health and social services to immigrant elders: The Islington Study.
- Lorek et al. (2009). The mental and physical health difficulties of children held within a British immigration detention center: A pilot study.
- Mahtani and Huq (1993). The use of a Western model across cultures.
- Mantovani and Thomas (2014). Choosing motherhood: The complexities of pregnancy decision-making among young black women 'looked after' by the State.
- Marvin-Dowle and Soltani (2023). Inter-generational differences in perinatal health behaviours: A secondary analysis of the born in Bradford cohort, disentangling ethnicity and migration.
- Mateus et al. (2022). Rates of depressive and anxiety symptoms in the perinatal period during the COVID-19 pandemic: Comparisons between countries and with pre-pandemic data.
- Maynard et al. (2012). Trends in suicide among migrants in England and Wales 1979-2003.
- McGranahan et al. (2024). Preconception health among migrant women in England: A cross-sectional analysis of maternity services data 2018-2019.
- Milgram and Zucker (1985). Congruent stress-preventing factors and anxiety in a long-term stress situation.
- Mladovsky (2023). Mental health coverage for forced migrants: Managing failure as everyday governance in the public and NGO sectors in England.
- Modrowski et al. (2021). Victimization profiles in girls involved in the juvenile justice system: A latent class analysis.
- Mohnani et al. (2023). An integrated approach to address UASC mental health-a pilot service improvement project in London.
- Moltrecht et al. (2025). Social connection and its prospective association with adolescent internalising and externalising symptoms: An exploratory cross-country study using retrospective harmonisation.
- Montoya-Arenas et al. (2022). Effect of exposure to socio-political violence and abuse during childhood on executive planning in adult life.
- Mood et al. (2017). The mental health advantage of immigrant-background youth: The role of family factors.
- Moore et al. (2019). Migration, ethnicity and mental health: Evidence from mothers participating in the Millennium Cohort Study.
- Morgan (2012). Aesop-10: A 10-year follow-up study of first-episode psychosis-rationale, method, and preliminary findings.
- Morgan (2012). Psychotic experiences in the general population and ethnicity: Findings from the SELCoH study.
- Morgan et al. (1975). The urban distribution of non-fatal deliberate self-harm.
- Morris et al. (2017). How do moving and other major life events impact mental health? A longitudinal analysis of UK children.
- Mudyarabikwa et al. (2022). Refugee and Immigrant Community Health Champions: A Qualitative Study of Perceived Barriers to Service Access and Utilisation of the National Health Service (NHS) in the West Midlands, UK.
- Mulvaney-Day et al. (2011). Preferences for relational style with mental health clinicians: A qualitative comparison of African American, Latino and Non-Latino White patients.
- Murphy et al. (2020). Healthcare access for children and families on the move and migrants.
- Myers and Neal (1978). Suicide in psychiatric patients.
- Nath et al. (2020). Strategies for recruiting and preventing drop-out of women with complex needs across the Effectiveness of services for Mothers with Mental Illness (ESMI) programme.
- Ndetei (1988). Psychiatric phenomenology across countries: Constitutional, cultural, or environmental?
- Neeleman et al. (1997). Suicide by age, ethnic group, coroners' verdicts and country of birth. A three-year survey in inner London.
- Nguyen et al. (2022). The mental health burden of racial and ethnic minorities during the COVID-19 pandemic.
- Nichols et al. (2022). Machine learning-based predictive modeling of resilience to stressors in pregnant women during COVID-19: A prospective cohort study.
- Niu et al. (2024). Age exacerbates the negative effect of depression on executive functioning in racial and ethnic minorities.
- Nyashanu et al. (2018). Experiences of first time mothers with postnatal depression among black sub-Sahara African communities in the West Midlands, England.
- Nyiri and Eling (2012). A specialist clinic for destitute asylum seekers and refugees in London.
- O'Reilly et al. (2021). Social variations in uptake of disability benefits: A census-based record linkage study.
- O'Shea et al. (2000). A school-based mental health service for refugee children.
- Ollawa et al. (2025). Occupational stress and wellbeing: A qualitative exploration of the perspectives and experiences of migrant sonographers practising in the United Kingdom.
- Oram (2021). Responding to the mental health needs of trafficked women.
- Ougrin et al. (2011). Suicide survey in a London borough: Primary care and public health perspectives.
- Papageorgiou et al. (2020). Patient data-sharing for immigration enforcement: A qualitative study of healthcare providers in England.
- Patch (1970). Homeless men: A London survey.
- Patel and Kull (2011). Assessing psychological symptoms in recent immigrant adolescents.
- Patel et al. (2023). Health needs of UASC and service provided by specialist nurses-awareness perspective audit of children and professionals in North West England.
- Pavarini et al. (2023). iSupport for Dementia: An analysis of clinical trial records.
- Petretti et al. (2013). Is violence a cause and consequence of HIV for women in England? A user-led mixed-method national stakeholder investigation.
- Picarda and Ineichen (1995). The mental health of ethnic minorities in the United Kingdom.
- Polling et al. (2019). Understanding geographical patterning of self-harm prevalence within a diverse urban population: A mixed methods spatial analysis and qualitative study.
- Power and Baxter (2024). The lived experience of food insecurity and mental health among undocumented migrants in the UK.
- Power et al. (2013). Developmentally and culturally appropriate screening in primary care: development of the behavioral health checklist.
- Priest (1970). Homeless men. A USA-UK comparison.
- Rack (1977). Some practical problems in providing a psychiatric service for immigrants.
- Rack (1980). Ethnic differences in depression and its response to treatment.
- Rack (1988). Psychiatric and social problems among immigrants.
- Ran et al. (2024). Negative wealth shocks and subsequent depressive symptoms and trajectories in middle-aged and older adults in the USA, England, China, and Mexico: A population-based, multinational, and longitudinal study.
- Rayment-Jones et al. (2017). A multisite audit to assess how women with complex social factors access and engage with maternity services.
- Redshaw et al. (2016). Impact of holding the baby following stillbirth on maternal mental health and well-being: Findings from a national survey.
- Resera et al. (2015). Interpreting in mental health, roles and dynamics in practice.
- Rhead et al. (2020). Impact of workplace discrimination and harassment among National Health Service staff working in London trusts: Results from the TIDES study.
- Rhead et al. (2022). A comparison of single and intersectional social identities associated with discrimination and mental health service use: Data from the 2014 Adult Psychiatric Morbidity Survey in England.
- Richardson et al. (2020). Sociodemographic inequalities in depression among older adults: Cross-sectional evidence from 18 countries.
- Rivas et al. (2025). Intersecting factors of disadvantage and discrimination and their effect on daily life during the coronavirus pandemic: The CICADA-ME mixed-methods study.
- Rivas et al. (2025). UK health and social care use during and after the pandemic: A qualitative study of the experiences of disabled people from minoritised ethnic groups.
- Robinson et al. (2025). Mechanisms and attitudes in responsive healthcare for forced migrant communities: A qualitative study of transnational practice.
- Robjant et al. (2009). Psychological distress amongst immigration detainees: A cross-sectional questionnaire study.
- Rogers-Sirin (2013). Segmented assimilation and attitudes toward psychotherapy: A moderated mediation analysis.
- Rosen et al. (2012). Interpersonal complementarity in the mental health intake: A mixed-methods study.
- Rudell et al. (2010). What effect do acculturative processes have on perception? A study on the concepts of severe distress amongst migrants and non-migrants in London.
- Ruiz et al. (2021). A cross-cultural exploratory study of health behaviors and wellbeing during COVID-19.
- Ruiz-Burga and Benoit (2021). Implications of migration patterns and sex work on access to health services and key health outcomes: A qualitative study on male migrant sex workers in London.
- Russell Jonsson et al. (2022). Health behaviors and subsequent mental health problems during the COVID-19 pandemic: A longitudinal analysis of adults in the UK.
- Safdar et al. (2007). Acculturation specific and general hassles and positive psychological functioning.
- Sala et al. (2021). Distance assessment of cognitive deficits in older immigrants.
- Sallah and Kozlowska (2015). People with learning disabilities from migrant communities, living in England: Improving access, experience and outcomes.
- Sangha et al. (2023). A qualitative study investigating the experiences of young adults caring for a sibling with disability within immigrant families in the UK: "Challenges are just the constant".
- Saunders et al. (2021). Healthcare utilization among migrants to the UK: cross-sectional analysis of two national surveys.
- Sen et al. (2018). Mental health morbidity among people subject to immigration detention in the UK: A feasibility study.
- Shah et al. (2009). Comparison of elderly suicide rates among migrants in England and Wales with their country of origin.
- Silveira and Ebrahim (1998). Social determinants of psychiatric morbidity and well-being in immigrant elders and whites in east London.
- Silveira et al. (2021). Ethnicity and parental discipline practices: A cross-national comparison.
- Sime (2014). 'I think that Polish doctors are better': Newly arrived migrant children and their parents' experiences and views of health services in Scotland.
- Simon et al. (2022). Contextualizing the well-being of asexual youth: Evidence of differences in family, health, and school outcomes.
- Smyth et al. (2015). Social networks, social support and psychiatric symptoms: Social determinants and associations within a multicultural community population.
- Stagg et al. (2022). Long-term health conditions in UK working-age adults: A cross-sectional analysis of associations with demographic, socioeconomic, psychosocial and health-related factors in an inner-city population.
- Stauffer et al. (2011). Trajectories of response to treatment with atypical antipsychotic medication in patients with schizophrenia pooled from 6 double-blind, randomized clinical trials.
- Steare et al. (2024). Economic inequalities in adolescents' internalising symptoms: Longitudinal evidence from eight countries.
- Stephan et al. (2024). Personality nuances and risk of dementia: Evidence from two longitudinal studies.
- Stevens et al. (2015). An Internationally Comparative Study of Immigration and Adolescent Emotional and Behavioral Problems: Effects of Generation and Gender.
- Stiles et al. (2021). Researching the Effectiveness of Tree of Life: An Imbeleko Approach to Counseling Refugee Youth.
- Subramanian et al. (2018). Retrospective data collection research and audit of services at a clinic for survivors of Female Genital Mutilation (FGM) in East London, England.
- Sugden (2021). Jiu-Jitsu and society: Male mental health on the mats.
- Szmukler (1981). Compulsory admissions in a London borough: II. Circumstances surrounding admission: Service implications.
- Tahira and Agius (2012). Epigenetics and migration - Considerations based on the incidence of psychosis in south Asians in Luton, England.
- Taylor et al. (2025). Trajectories of physical, mental health and health satisfaction before and after arthritis diagnosis: A UK population-based study.
- Tham et al. (2023). Suicide among psychiatric patients who migrated to the UK: A national clinical survey.
- Tompkins et al. (2003). Associations between migrancy, health and homelessness: A cross-sectional study.
- Torres Stone et al. (2020). A Community Mental Health Needs Assessment of a Racially and Ethnically Diverse Population in New England: Narratives from Community Stakeholders.
- Trabold et al. (2013). An exploratory analysis of intimate partner violence and postpartum depression in an impoverished urban population.
- Tunstall et al. (2010). Residential mobility in the UK during pregnancy and infancy: Are pregnant women, new mothers and infants 'unhealthy migrants'?
- Tunstall et al. (2015). Difficult Life Events, Selective Migration and Spatial Inequalities in Mental Health in the UK.
- Turkoglu et al. (2020). The workplace meal: A migrant workers' perspective.
- Upadhyaya and Creed (1990). Psychiatric morbidity among mothers attending well-baby clinic: A cross-cultural comparison.
- Vandrevala et al. (2017). Perceived needs for support among care home staff providing end of life care for people with dementia: A qualitative study.
- Verelst et al. (2022). Social determinants of the mental health of young migrants.
- Verity and Brown (2023). Inclusion health patient perspectives on remote access to general practice: A qualitative study.
- Walker et al. (2021). Mental health of forced migrants recently granted leave to remain in the United Kingdom.
- Wang and Giovanis (2024). Cultural integration, subjective identity, and well-being: Global migrants in the UK.
- Ward et al. (2003). Lost and found: Reinvention of the self following migration.
- Watson and Evans (1986). An example of cross-cultural measurement of psychological symptoms in post-partum mothers.
- White (1974). Self-poisoning in adolescents.
- Whitley and Zhou (2020). Clueless: An ethnographic study of young men who participate in the seduction community with a focus on their psychosocial well-being and mental health.
- Wicks et al. (2024). Are protected characteristics associated with mental health care inequalities in the adult UK general population? A cross-sectional study
- Wilding et al. (2018). Place and preference effects on the association between mental health and internal migration within Great Britain.
- Wright and Thomas (2020). Pre-discharge factors associated with early readmission to psychiatric inpatient services within 90 days
- Wu et al. (2018). Acculturation, resilience, and the mental health of migrant youth: A cross-country comparative study.
- Zhang et al. (2022). Migrants' primary care utilisation before and during the COVID-19 pandemic in England: An interrupted time series
- Zivin et al. (2010). Depression among older adults in the United States and England.
- Ziyachi and Castellani (2024). A 'Cultural Models' Approach to Psychotherapy for Refugees and Asylum Seekers: A Case Study from the UK.
- van Lent et al. (2025). Evaluating an intervention to promote access to mental healthcare for low language proficient migrants and refugees across Europe (MentalHealth4All): study protocol for a pretest-post-test cross-national survey study.

*Results not differentiated for Latin Americans (n = 21)*

- Bhui et al. (2025). Racialised experience of detention under the Mental Health Act: A photovoice investigation.
- Bramley et al. (2020). Exploring the support for UK migrants experiencing gambling-related harm: Insights from two focus groups.
- Clark et al. (2022). Quantifying the health needs of migrants in vulnerable circumstances registered with a nurse-led primary care service.
- Croak et al. (2025). Inequalities in NHS Staff Support among those from Ethnic Minority and Migrant groups during the COVID-19 Pandemic.
- Garcia-Medrano and Panhofer (2020). Improving migrant well-being: Spontaneous movement as a way to increase the creativity, spontaneity and welfare of migrants in Glasgow.
- Hatch et al. (2016). Discrimination and common mental disorder among migrant and ethnic groups: Findings from a South East London Community sample.
- Hollins et al. (2011). A demographic survey to identify aspects of lifestyle, conditions and characteristics of London sex-workers.
- Howard and Hodes (2000). Psychopathology, adversity, and service utilization of young refugees.
- Kessler et al. (2020). Reducing Intrusive Memories of Trauma Using a Visuospatial Interference Intervention With Refugees With Posttraumatic Stress Disorder (PTSD): A Test of Replication to a New Population.
- Lynam et al. (2007). Understanding marginalization as a social determinant of health.
- McLeish and Redshaw (2017). Mothers' accounts of the impact on emotional wellbeing of organised peer support in pregnancy and early parenthood: A qualitative study.
- Ngwena and Aisenberg (2014). Black and minority ethnic groups (BME) suicide, admission with suicide or self-harm: An inner city study.
- Orton et al. (2012). Resilience among asylum seekers living with HIV.
- Owuamalam et al. (2017). Socially creative appraisals of rejection bolster ethnic migrants' subjective well-being.
- Palmer and Ward (2007). 'Lost': Listening to the voices and mental health needs of forced migrants in London.
- Rafighi et al. (2016). National Health Service Principles as Experienced by Vulnerable London Migrants in "Austerity Britain": A Qualitative Study of Rights, Entitlements, and Civil-Society Advocacy.
- Reyes (1989). The destruction of the soul: A treatable disease.
- Ruiz-Burga (2021). Implications of Migration Patterns and Sex Work on Access to Health Services and Key Health Outcomes: A Qualitative Study on Male Migrant Sex Workers in London.
- Sun et al. (2020). Never Mind the Acculturation Gap: Migrant Youth's Wellbeing Benefit when They Retain Their Heritage Culture but Their Parents Adopt the Settlement Culture.
- Sun et al. (2024). Home is where the heart is: Implications of dyadic acculturation for migrant couples' personal and relational well-being.
- Uskul et al. (2024). Honour, acculturation and well-being: Evidence from the UK and Canada.

*Outcomes or topics not relevant or covered in sufficient depth (n = 46)*

- Asif et al. (2022). Structural barriers to refugee, asylum seeker and undocumented migrant healthcare access. Perceptions of Doctors of the World caseworkers in the UK.
- Bagley et al. (1973). Social structure and the ecological distribution of mental illness, suicide, and delinquency.
- Benitez et al. (2019). An Integrated Approach to Bias in a Longitudinal Survey in the United Kingdom: Assessing Construct, Method, and Item Bias in the General Health Questionnaire (GHQ-12).
- Ceuterick et al. (2011). Resilience of Andean urban ethnobotanies: A comparison of medicinal plant use among Bolivian and Peruvian migrants in the United Kingdom and in their countries of origin.
- Coren et al. (2016). Interventions for promoting reintegration and reducing harmful behaviour and lifestyles in street-connected children and young people.
- Davies et al. (2013). Hospital activity and cost incurred because of unregistered patients in England: Considerations for current and new commissioners.
- Davis et al. (2019). Economic vulnerability, cultural decline, and nativism: Contingent and indirect effects.
- Eisen et al. (2023). Infections in Asymptomatic Unaccompanied Asylum-seeking Children in London 2016-2022.
- Erisen and Vasilopoulou (2022). The affective model of far-right vote in Europe: Anger, political trust, and immigration.
- Forbat (2025). Financial insecurity for terminally ill migrants in the UK: Double jeopardy at the end of life.
- Furuya et al. (2023). Understanding Internal Migration: A Research Note Providing an Assessment of Migration Selection With Genetic Data.
- Gerayeli and Kyriakopoulos (2025). How do therapists in the UK navigate the issue of loneliness when working with destitute asylum seekers? A focus group using reflexive thematic analysis.
- Gideon J. (2011). The health and well-being of Latin American migrants in London.
- Golec de Zavala et al. (2017). The relationship between the Brexit vote and individual predictors of prejudice: Collective narcissism, right wing authoritarianism, social dominance orientation.
- Hall (1968). Future of the population of north-east Scotland: A statistical study.
- Hartman et al. (2021). The authoritarian dynamic during the COVID-19 pandemic: Effects on nationalism and anti-immigrant sentiment.
- Hill et al. (2011). Culture and sleep: How do sleep habits compare between high and low altitude dwelling bolivian children and a low land northern european population?
- Kelley et al. (2025). 'They have somewhere to turn to': Wellbeing support for newly arrived refugee and migrant adolescents in English secondary schools.
- Kerr et al. (2024). The Missing Voices, an exploration of what healthcare-related research would matter to Global Ethnic Majority Women in Aberdeenshire, Scotland.
- Kozhevnikov (2021). Career capital in global versus second-order cities: Skilled migrants in London and Newcastle.
- Llop-Girones et al. (2021). Employment and working conditions of nurses: Where and how health inequalities have increased during the COVID-19 pandemic?
- Lloyd et al. (2021). "Just tensions left, right and centre": Assessing the social impact of international migration on deindustrialized locale.
- Malla et al. (2014). Facing new challenges in the evolution of early intervention services: Research and an agenda for action.
- Marozzi and Aiello (2016). Construction, robustness assessment and application of an index of perceived level of socio-economic threat from immigrants: A study of 47 European countries and regions.
- Mas Giralt and Granada (2015). Latin Americans migrating from Europe to the UK: Barriers to accessing public services and welfare.
- Miller et al. (2024). Depressive Symptoms, Socioeconomic Position, and Mortality in Older People Living with and beyond Cancer.
- Mladovsky (2009). A framework for analysing migrant health policies in Europe.
- Namata and Hatzidimitriadou (2023). Strategies for improving access to primary care services for homeless immigrants in England: A Delphi study.
- Nicholls et al. (2022). Experiences of and attitudes towards HIV testing for Asian, Black and Latin American men who have sex with men (MSM) in the SELPHI (HIV Self-Testing Public Health Intervention) randomized controlled trial in England and Wales: Implications for HIV self-testing.
- Ngwena (2014). Black and minority ethnic groups (BME) suicide, admission with suicide or self-harm: An inner city study.
- Oduola et al. (2025). Changing the trajectories of mental health difficulties in Norfolk and Suffolk: A research-priority-setting project with patients, the public, clinicians, policymakers and other stakeholders-study protocol.
- Paralikar et al. (2009). Diagnosing chronic fatigue syndrome.
- Pierce et al. (2025). Effects of UK hostile environment policies on maternity care for refugees, asylum seekers, and undocumented migrants in Camden: Examining the experiences of healthcare professionals and community organisations.
- Riley et al. (2023). A mixed methods evaluation of a community health and wellbeing worker pilot in three settings in England.
- Rose-Clarke et al. (2020). Rethinking research on the social determinants of global mental health.
- Ruiz et al. (2021). The anxiety of political uncertainty: Insights from the Brexit vote.
- Schiefer et al. (2024). Science and Heritage Language Integrated Learning (SHLIL): Evidence of the effectiveness of an innovative science outreach program for migrant students.
- Scuffell et al. (2024). Incidence of long-term conditions in the Latin American community of London: A validation and retrospective cohort study of 890,922 primary care records, 2005-2022.
- Silva et al. (2025). Experiences of antibiotic use and healthcare access among migrants to the UK: A qualitative study.
- Singh and Sen (2018). From reflection to action: A multimodal, interactive approach to enhancing training on child refugee health.
- Siva (2010). Stopping traffic.
- Smithman et al. (2020). Typology of organizational innovation components: Building blocks to improve access to primary healthcare for vulnerable populations.
- Spice and Anon (2007). Management of sex workers and other high-risk groups.
- Sweileh (2018). Global output of research on the health of international migrant workers from 2000 to 2017.
- Wallace (2018). Policy-driven evidence: Evaluating the UK government's approach to immigration policy making.
- Wood and Devakumar (2020). Healthcare access for migrant children in England during the COVID-19 pandemic
- van Tilburg et al. (2021). Citizen vain? Exposure to the UK citizenship test predicts milder demands from immigrants across the political spectrum.

*Not primary research (n = 155)*

- Adams et al. (2024). Leading a community health & wellbeing service in a community NHS trust.
- Agius and Ward (2009). The epidemiology of psychosis in Luton.
- Alcock (2003). Refugee trauma - The assault on meaning
- Allen (2013). The ethical dilemmas associated with working in an immigration removal centre.
- Anderson et al. (2020). Migration and perinatal mental disorders: A systematic review & meta-analysis, and findings from the WENDY study.
- Angeli et al. (2025). "This Should Be the Answer!": The Evolution of Relational Dynamic Capabilities in the Co-Production of Maternity Care Services to Vulnerable Women.
- Ani (2007). Working with asylum seekers with mental illness distressed by the home office dispersal programme.
- Appleby et al. (2017). New policy and evidence on suicide prevention.
- Awofeso and Applebaum (2010). Preventing suicides in prison settings-The role of mental health promotion policies and programs.
- Badat (2010). Ethnic minorities and mental health services: Developing a more sustainable approach.
- Bagley (1971). The social aetiology of schizophrenia in immigrant groups.
- Baubet et al. (2009). Psychotic states in young migrants and children of migrants.
- Bhugra (2005). Cultural identities and cultural congruency: A new model for evaluating mental distress in immigrants.
- Bhugra (2019). Migration, violence, and the role of psychiatry in Europe.
- Bhugra et al. (2017). Acculturation, violent radicalisation, and religious fundamentalism.
- Bhui (2011). A debate on cultural competence in mental health is urgently needed.
- Bhui (2018). Tackling ethnic inequalities in severe mental illness experiences and outcomes.
- Biesty et al. (2024). Community led health promotion to counter stigma and increase trust amongst priority populations: Lessons from the 2022-2023 UK mpox outbreak.
- Birmingham City Council (2024). Latin American Community Mental Health Profile.
- Boukari and Devakumar (2024). Far-right riots in the UK: The culmination of years of rhetoric and policies.
- Bragg (2008). Maternal deaths and vulnerable migrants.
- Brenman (2021). Placing precarity: Access and belonging in the shifting landscape of UK mental health care.
- British Medical Journal (1973). Rootless wanderers.
- Brown and Levinson (2022). Core eating disorder fears: Prevalence and differences in eating disorder fears across eating disorder diagnoses.
- Budd (2023). Supporting Communities in Humanitarian Crises with Acupuncture and Integrative Medicine A Perspective.
- Burvill (1973). Immigration and mental disease.
- Butler et al. (2015). Migration and common mental disorder: An improvement in mental health over time?
- Carignan and Iseman (2004). Contrasting clinical techniques: A British Kleinian, contemporary Freudian and Latin American Kleinian discuss clinical material.
- Chaplin et al. (2020). Refugee mental health research: Challenges and policy implications.
- Cheli et al. (2016). Mediterrean bridges and drifts: The Ruropean migrant crisis and the role of psychooncology.
- Christensen and Manthorpe (2016). Personalised risk: New risk encounters facing migrant care workers.
- Cohen (2010). Asylum seekers' health needs. With what right do we detain?
- Cooper (2005). Schizophrenia, social class and immigrant status: The epidemiological evidence.
- Crafa and Warfa (2015). Maternal migration and autism risk: Systematic analysis.
- Craig et al. (2006). Mental healthcare needs of refugees.
- Crawford et al. (2011). The health and well-being of remote and mobile workers.
- Crawshaw and Kirkbride (2018). Public Health England's Migrant Health Guide: An online resource for primary care practitioners.
- Das-Munshi et al. (2013). Does childhood adversity account for poorer mental and physical health in second-generation Irish people living in Britain? Birth cohort study from Britain (NCDS).
- De et al. (2005). Epidemiology of bipolar disorders.
- Dean (2011). Highlights of this issue.
- Devakumar and Issa (2022). Migrant traumas heard.
- Dexter and Katona (2018). Hunger strike renews concerns over health in UK detention centres.
- Dikaiou et al. (2010). Prologue.
- Duarte et al. (2024). Who belongs? Immigration, ethnicity, and mental health.
- Dykxhoorn et al. (2024). Hiding in plain sight: Ethnic and migrant variation in suicide.
- Escobar (2012). Diagnostic bias: Racial and cultural issues.
- Faden et al. (2022). Who to vaccinate first? A peek at decision-making in a pandemic.
- Fair et al. (2025). Parent's experiences of the impact of ethnicity and skin pigmentation on perinatal care.
- Fazel and Stein (2002). The mental health of refugee children.
- Feliciano Graniela et al. (2024). Identifying the effects of racism and coloniality through the power, threat, and meaning framework.
- Fisher (2009). Film on NHS for new immigrants.
- Fledderjohann et al. (2021). Mental health and care needs of British children and young people aged 6-17.
- Flensmark (2004). Is there an association between the use of heeled footwear and schizophrenia?
- Fung et al. (2006). Ethnicity and mental health: the example of schizophrenia in migrant populations across Europe.
- Fung et al. (2009). Ethnicity and mental health: The example of schizophrenia and related psychoses in migrant populations in the Western world.
- Gariepy et al. (2015). Measuring the impact of pregnancy intention on women's health-related quality of life.
- Ghaffari (2011). Immigration and postnatal depression.
- Ginapp et al. (2023). The experiences of adults with ADHD in interpersonal relationships and online communities: A qualitative study.
- Giscombe et al. (2020). Perinatal mental health amongst refugee and asylum-seeking women in the UK.
- Glover (1991). The use of inpatient psychiatric care by immigrants in a London Borough.
- Goldenberg (2015). Supporting the mental health of trafficked people.
- Goldwyn (2013). Many detainees shouldn't be there in the first place.
- Grant-Peterkin (2018). Mental health needs in immigration removal centres.
- Hashmi (1970). Discussion on problems of an immigrant population. Immigrants and emotional stress.
- Hatch et al. (2020). Intersectional Social Identities, Discrimination And Mental Health Service: Findings From The 2014 Adult Psychiatric Morbidity Survey Of England.
- Heald et al. (2018). Brexit, the Leave campaign, and mental health of ethnic minority communities.
- Heald et al. (2018). The LEAVE vote and racial abuse towards Black and Minority Ethnic communities across the UK: The impact on mental health.
- Healthwatch Southwark (2024). Overview of Responses to Healthwatch Southwark’s Latin American Access Report.
- Hiam and Mckee (2016). Making a fair contribution: Is charging migrants for healthcare in line with NHS principles?
- Hiam et al. (2018). Creating a 'hostile environment for migrants': The British government's use of health service data to restrict immigration is a very bad idea.
- Hiam et al. (2019). Overcoming the barriers migrants face in accessing health care.
- Hitch (1977). Culture, social structure, and the explanation of migrant mental illness.
- Humphrey et al. (2025). GP remote consultations with marginalised patients and the importance of place during care: A qualitative study of the role of place in GP consultations.
- Jackson et al. (2012). Immigrant-native differences in child health: Does maternal education narrow or widen the gap?
- Jarvis (1998). Schizophrenia in British immigrants: Recent findings, issues and implications.
- Kellezi et al. (2021). Adapting Social Prescribing to meet the needs of migrant populations: Challenges and solutions to service access and efficacy.
- Kiddo et al. (2022). COVID-19 pandemic impact on unaccompanied asylum-seeking children (UASC) tuberculosis screening service.
- Kirby et al. (2024). 52682 Revealing patient perspectives on hidradenitis suppurativa according to racial background: Results from a global study.
- Knies et al. (2016). Life satisfaction, ethnicity and neighbourhoods: Is there an effect of neighbourhood ethnic composition on life satisfaction?
- Knights et al. (2022). Initial health assessments for newly arrived migrants, refugees, and asylum seekers
- Lewis (1963). Demographic aspects of mental disorder.
- Lipsedge and Turner (1990). Services for migrant drug abusers.
- Littlewood (1985). The migration of culture-bound syndromes.
- Livingston and Sembhi (2003). Mental health of the ageing immigrant population.
- Lonergan (2024). Reproduction and the expanding border: Pregnant migrants as a 'problem' in the 2014 Immigration Act.
- Maffla (2008). Health in the age of migration: Migration and health in the EU.
- Mandaglio and Maiera (2014). Psychosis and migration: Does a correlation exist?
- March et al. (2010). Psychosis in migrant and minority populations: Prescriptions for scientific and social policy.
- Marmorstein (2017). Sleep patterns and problems among early adolescents: Associations with alcohol use.
- McKenzie (2010). Building consensus for moving forward.
- Mengistu and Manolova (2019). Acculturation and mental health among adult forced migrants: A meta-narrative systematic review protocol.
- Miller (1977). Transcultural psychiatry: From the Proceedings of the International Congress on Transcultural Psychiatry, Bradford, July 1976.
- Mladovsky (2024). Reproducing differential racialisation: Social triage in forced migrant mental health services in England.
- Morisaki (2022). Understanding and reducing perinatal health disparities for immigrants and between ethnicities.
- Muraleetharan and Saunders (2025). Understanding associations between sexual identity change and the mental health of lesbian, gay, and bisexual adults in the United Kingdom through longitudinal survey.
- O'Brien (2007). Refugee and migrant vulnerability to injury.
- Oduola et al. (2021). Change in incidence rates for psychosis in different ethnic groups in south London: Findings from the Clinical Record Interactive Search-First Episode Psychosis (CRIS-FEP) study.
- Ohlund et al. (2018). Conference proceedings of the 4th Masterclass Psychiatry: Transcultural Psychiatry-Diagnostics and Treatment.
- Ozturk et al. (2024). A Bibliometric Analysis of Refugee Health Publications in the Nursing Field by Visual Mapping Method.
- Palmer (2007). Face to face.
- Patel et al. (2021). Unmet need for mental health medication within the migrant population of Northern Ireland: A record linkage study.
- Pereda et al. (2012). A case study perspective on psychological outcomes after female genital mutilation.
- Philipp (2010). Public health support for refugees and asylum seekers.
- Pickles and Hartree (2013). Transferring healthcare for immigration detainees in England to the NHS: Should improve services, particularly for those with mental illness.
- Procter et al. (2010). Editorial: Global economic crises and mental health.
- Puthussery (2016). Perinatal outcomes among migrant mothers in the United Kingdom: Is it a matter of biology, behaviour, policy, social determinants or access to health care?
- Razai et al. (2021). Mitigating ethnic disparities in covid-19 and beyond
- Redshaw et al. (2016). The impact of holding the baby following stillbirth on maternal mental health and wellbeing: Findings from a national survey.
- Rivas et al. (2024). Compliance with COVID-19 government guidance and rules by disabled people and people from minoritised ethnic groups: Qualitative findings from the CICADA study.
- Rock et al. (2008). The seasonal risk for deliberate self-harm.
- Rollin (1972). Has the mental hospital got a future?
- Rutter et al. (1975). Attainment and adjustment in two geographical areas. I--The prevalence of psychiatric disorder.
- Skone (1963). Prevention of psychiatric disorders among immigrant schoolchildren in Britain.
- Sanchez Clemente et al. (2022). A Whole-child, whole-family approach to health assessments for asylum-seeking children.
- Sandhu (2011). Child and adolescent mental health services in multicultural Britain, hopes and challenges.
- Saravane et al. (2009). Drawing up guidelines for the attendance of physical health of patients with severe mental illness.
- Schmachtenberg et al. (2020). How is migration background considered in the treatment and care of people? A comparison of national dementia care guidelines in Europe.
- Seidel (1966). A study of mental subnormality in childhood.
- Selten et al. (2007). Migration and schizophrenia.
- Sen et al. (2014). Mental health care for foreign national prisoners in England and Wales.
- Shah (2007). Can the recognition of clinical features of mental illness at clinical presentation in ethnic elders be improved?
- Shur (1982). Family history and schizophrenia: Characteristics of groups with and without positive family histories.
- Sim and Mackie (2018). Well-being of the migrant workforce: Who cares?
- Singh (2009). Response to 'The social determinants of psychosis in migrant and ethnic minority populations: A public health tragedy'.
- Sobotka and Ross (2023). Newborn Screening for Neurodevelopmental Disorders May Exacerbate Health Disparities.
- Spaas et al. (2023). School-based Psychosocial Interventions' Effectiveness in Strengthening Refugee and Migrant Adolescents' Mental Health, Resilience, and Social Relations: A Four-country Cluster Randomized Study.
- Stevens (2020). How can we meet the health needs of child refugees, asylum seekers and undocumented migrants?
- Straw et al. (2018). Sexual health of female asylum-seeking teenagers: Identifying knowledge gaps, tackling perceived barriers, and suggestions for intervention.
- Sweileh (2023). Analysis and Mapping of Scientific Literature on Detention and Deportation of International Migrants (1990-2022).
- Swinnen et al. (2007). Mood disorders and migration. Meta-analysis.
- Teles and Viana (2021). Science outreach in my mother tongue.
- The Lancet (1972). Emotional stress in immigrant workers.
- The Lancet (1990). Mental health services for migrants in Europe.
- The Lancet, Rheumatology (2025). Inequality and health: All is not equal.
- Till et al. (2019). The mental health of foreign national prisoners.
- Tomkow et al. (2020). Healthcare access for asylum seekers and refugees in England: A mixed methods study exploring service users' and health care professionals' awareness.
- Toniolo (2007). Psychiatric disorders in a transcultural setting.
- Tribe and Antinucci (2019). Using co-production within mental health training when working with refugee or migrant community groups.
- Tribe and Farsimadan (2022). Guidance for clinicians when working with refugees and asylum seekers.
- Tribe and Thompson (2022). Working with interpreters when working with forced migrants in mental health.
- Tribe et al. (2011). Developing guidelines on working with interpreters in mental health: Opening up an international dialogue?
- Tunstall et al. (2014). The general and mental health of movers to more- and less-disadvantaged socio-economic and physical environments within the UK.
- Tunstall et al. (2015). Residential mobility and the association between physical environment disadvantage and general and mental health.
- Vacchelli and Al-Hindi (2018). Embodiment in qualitative research: Collage making with migrant, refugee and asylum seeking women.
- Van Hout et al. (2020). Migrant health situation when detained in European immigration detention centres: a synthesis of extant qualitative literature.
- Veling (2013). Ethnic minority position and risk for psychotic disorders.
- Verdonk (1979). Migration and mental illness.
- Vicente et al. (1993). Users' satisfaction with mental health services.
- Vo and Canty (2023). Global mental health experiences of single mothers: A mixed methods research synthesis.
- Vogel-Scibilia (2015). Finding Hannah.
- Voracek and Loibl (2008). Consistency of immigrant and country-of-birth suicide rates: A meta-analysis.
- Waterman and Katona (2020). Assessing asylum seekers, refugees and undocumented migrants.
- Waterman et al. (2022). The mass release of migrants from UK immigration detention during the COVID-19 pandemic: What can be learned?
- Watters (2007). Refugees at Europe's borders: The moral economy of care.
- Webb (1998). Children and the inverse care law.
- Wickramage and Mosca (2014). Can migration health assessments become a mechanism for global public health good?
- Wild et al. (2007). Mortality from all causes and circulatory disease by country of birth in England and Wales 2001-2003.
- Wintrob (2013). Reflections on current research and future challenges in cultural psychiatry.
- Wittkowski et al. (2017). The Experience of Postnatal Depression in Immigrant Mothers Living in Western Countries: A Meta-Synthesis.
- Wright and Brampton (2009). Going home: Migration as enactment and symbol.
- Zimmerman et al. (2009). Meeting the health needs of trafficked persons.
- von Humboldt et al. (2025). "Until COVID-19 do us part": A qualitative study on perceived sexual intimacy and mental health in heterosexual older couples during the Covid-19 pandemic.

*Full text unavailable (n = 8)*

- Cosh (2013). Improving healthcare access for difficult-to-reach groups.
- Gainsborough et al. (2023). Adverse childhood experiences of refugee and asylum-seeking children: Identifying risk and resilience factors for mental health and wellbeing.
- Hart et al. (2016). HIV care for Latin American patients in London: Recognising the diversity and complex needs of this minority ethnic group: A service evaluation.
- Liang et al. (2019). Impact of International Immigration on Life Satisfaction of Local Residents in England: Exploring the Differentiated Relationships across Socioeconomic Gradients.
- Mooren and Allen (2011). Parents and children together: Multifamily therapy for refugees with complex trauma symptoms.
- Nwaru et al. (2024). Longitudinal associations between nonstandard work schedule and depressive symptoms: Are there differences by sex, ethnicity, and immigrant status?
- Sumpter et al. (2024). Building belonging: The role of community cafes.
- Yan et al. (2019). Does Immigrant Generation Matter? Re-examining the Ethnic Density Effects on Mental Health of Ethnic Minorities in Britain.

*Not yet published (n = 3)*

- Drew and Hamana (2023). SParky Samba - Exploring the benefits of Samba percussion [ongoing study].
- Harris (2023). A Study to Assess the Effects of a Randomised Community Health Worker Intervention on Uptake of Preventative Care Services in London [ongoing study].
- University of Aberdeen (2024). Maternity Care Experiences of Asylum-seeking and Refugee Women in Scotland A1 [ongoing study].
